# MaxEnt Modeling to Predict the Current and Future Distribution of *Pomatosace filicula* under Climate Change Scenarios on the Qinghai–Tibet Plateau

**DOI:** 10.3390/plants11050670

**Published:** 2022-02-28

**Authors:** Kaiyang Chen, Bo Wang, Chen Chen, Guoying Zhou

**Affiliations:** 1 Northwest Institute of Plateau Biology, Chinese Academy of Sciences, Xining 810008, China; chenkaiyang@nwipb.cas.cn (K.C.); wangbo1949@126.com (B.W.); chenchen@nwipb.cas.cn (C.C.); 2 University of Chinese Academy of Sciences, Beijing 100049, China

**Keywords:** *Pomatosace filicula*, Qinghai–Tibet Plateau, climate change, MaxEnt model, potential distribution

## Abstract

As an important Tibetan medicine and a secondary protected plant in China, *Pomatosace filicula* is endemic to the country and is mainly distributed in the Qinghai–Tibet Plateau (QTP). However, global climate change caused by greenhouse gas emissions might lead to the extinction of *P. filicula*. To understand the potential spatial distribution of *P. filicula* in future global warming scenarios, we used the MaxEnt model to simulate changes in its suitable habitat that would occur by 2050 and 2070 using four representative concentration pathway (RCP) scenarios and five global climate models. The results showed that the QTP currently contains a suitable habitat for *P. filicula* and will continue to do so in the future. Under the RCP2.6 scenario, the suitable habitat area would increase by 2050 but shrink slightly by 2070, with an average reduction of 2.7%. However, under the RCP8.5 scenario, the area of unsuitable habitat would expand by an average of 54.65% and 68.20% by 2050 and 2070, respectively. The changes in the area of suitable habitat under the RCP4.5 and RCP6.0 scenarios were similar, with the unsuitable area increasing by approximately 20% by 2050 and 2070. Under these two moderate RCPs, the total suitable area in 2070 would be greater than that in 2050. The top three environmental factors impacting the habitat distribution were altitude, annual precipitation (BIO12) and annual temperature range (BIO7). The cumulative contribution rate of these three factors was as high as 82.8%, indicating that they were the key factors affecting the distribution and adaptability of *P. filicula*, *P. filicula* grows well in damp and cold environments. Due to global warming, the QTP will become warmer and drier; thus, the growing area of *P. filicula* will move toward higher elevations and areas that are humid and cold. These areas are mainly found near the Three-River Region. Future climate change will aggravate the deterioration of the *P. filicula* habitat and increase the species’ survival risk. This study describes the distribution of *P. filicula* and provides a basis for the protection of endangered plants in the QTP.

## 1. Introduction

Global climate change is a major challenge for humans and ecosystems in the 21st century [1]. The current concentration of greenhouse gases, especially carbon dioxide, has increased by 70% compared to 1970 [2]. Global warming is expected to continue, with the average Earth’s surface temperature rising by 0.3–4.5 °C degrees Celsius by 2100 compared to 1986 to 2005 [3,4]. Rising average temperatures pose a serious threat to the sustainability of global ecosystems and have already altered the biodiversity of landscapes worldwide [5,6]. Many studies have shown that species move to higher elevations or latitudes in response to a warming climate [7,8,9]. However, species’ climatic niches may change more slowly than climate change [10]. Thus, if future climate warming leads to the loss of suitable habitat or if geographical obstacles prevent the spread of species, some low-diffusion species will become endangered [11,12,13]. For example, Sony et al. [14] showed that as global warming intensifies, most of the suitable habitat for *Nilgiritragus hylocrius* will be lost. For instance, coffee agriculture in Ethiopia will probably be seriously affected by a warming climate, and the amount of land suitable for coffee production in low-altitude areas will be greatly reduced [15]. A recent simulation study in sub-Saharan Africa showed that up to 30% of corn and banana and up to 60% of bean agricultural areas are expected to be lost by the end of this century [16].

Species can change their niches through evolutionary adaptation [17]; however, species’ climatic niches may evolve more slowly than climate change [8,10], and failure to respond to changing abiotic and biotic conditions may lead to range shrinkage [8,18]. Species living in alpine regions are often highly sensitive to climate change [19,20]. Owing to increased competition for colder habitats and limited scope for expanding into new habitats, the increase in greenhouse gases and associated global warming are not conducive to the future success of alpine plants [8,21]. Therefore, plants in alpine areas are at a much greater risk of extinction than those that can grow in warmer environments and at low altitudes. Because these alpine species are extremely sensitive to climate change, it is anticipated that 60% of them will be lost [22]. The Himalayas Hengduan Mountains are representative of the alpine regions. A comparison of recent photos of climate change over the past few decades reveals the retreat of glaciers in the region, followed by the upward movement of the alpine tree line [23]. One study showed that in mountainous areas of Europe, with the increase in temperature, warm-adapted species have begun to migrate to higher elevations, resulting in the disappearance of cold-adapted species. *Cordyceps sinensis*, *Rhodiola*, *Saussurea involucrata*, *yew* and *Fritillaria* have all been found to be at risk of extinction [24,25].

**Figure 1:**
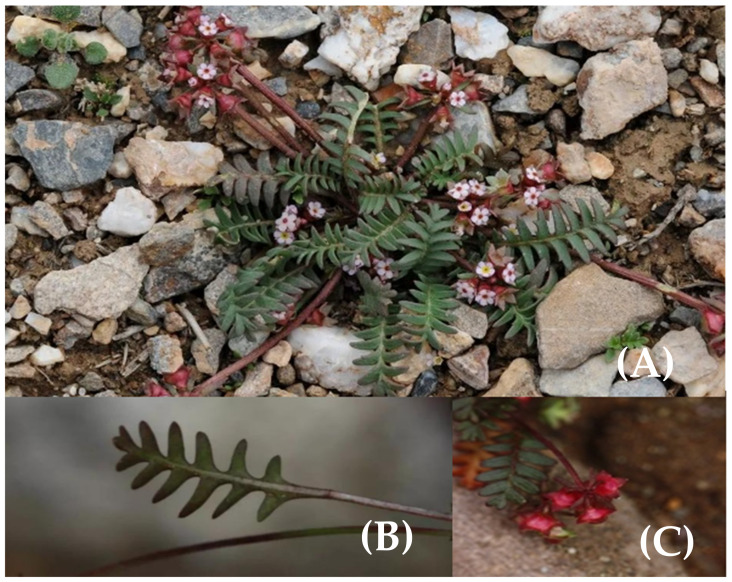
*Pomatosace filicula*: (**A**) in natural habitat; (**B**) leaf of *Pomatosace filicula*; (**C**) flowers of *Pomatosace filicula*. (Figure taken from ppbc.iplant.cn website (accessed on 6 October 2021), photographed by Yuhu Wu and Xinxin Zhu).

The Qinghai–Tibet Plateau (QTP) is located in the inland plateau of Asia, with an altitude of 3000–5000 m and an average altitude of more than 4000 m. It is known as the “roof of the world” and the “third pole.” Yw, Liang, Jw, Ww, Nn, Yg and Xw [25] predicted that the annual average temperature rise in the QTP will be 1.6 °C to 2.0 °C by 2050 due to global warming caused by greenhouse gas emissions. By the 2070s, the QTP’s annual average temperature will have risen by 1.2 °C to 3.0 °C. Many studies have shown that the continuous temperature rise threatens the growth of alpine crops on the QTP, and many species will be reduced in number and may face extinction [25,26]. *Pomatosace filicula* is a species in the family Primulaceae and has a 1–2 year growth cycle (Figure 1). It mainly grows in mountain basins at altitudes of 3200–4600 m and is distributed in Tibet, southern Gansu and Qinghai [27,28,29]. As a national secondary protected plant, *P. filicula* has important medicinal and economic value [30,31]. As a traditional Chinese medicine, *P. filicula* has analgesic and antipyretic effects and can be used to treat high blood pressure, uterine bleeding, irregular menstruation and arthritis [28,32,33,34]. Research on *P. filicula* has only focused on Tibetan medicine and genome sequencing, and there have been no studies on its distribution or factors that influence its habitat.

In this study, large bioclimatic variable datasets were combined in the maximum entropy (Maxent) model to simulate the future distribution patterns of *P. filicula*. The objectives of this study were: (1) to determine the area of suitable habitat and the most important environmental factors affecting the range of *P. filicula* under current climate scenarios; (2) to clarify how the suitable area will change as a result of global warming; and (3) to provide suggestions for the protection of *P. filicula.*

## 2. Results

### 2.1. Model Selection and Evaluation

The stability of the MaxEnt model was verified by 100 iterations of the jackknife method, and the prediction accuracy was evaluated by ROC curves. The test AUC and mean training AUC values obtained from the final model exceeded 0.9 for the current (Figure S1) and future (Figure S4) predictions. Because the current and future AUC values obtained by MaxEnt were greater than 0.9, the model simulation was found to be “excellent.” The model had high accuracy and could model the geographical distribution of *P. filicula* well.

### 2.2. Critical Environmental Factors

The MaxEnt jackknife test tool was used to evaluate the impact of various environmental variables on predicting the current (Figure S2) and future (Figure S5) distributions of *P. filicula*. The MaxEnt model automatically outputs the contribution rate of each variable. The variables alt, an, tk, ap, BIO1, BIO3, BIO4, BIO7, BIO12 and BIO15 were included in the final analysis. The contribution rates from high to low were as follows: altitude (52.4%), annual precipitation (15.3%), temperature annual range (BIO5-BIO6) (15.1%), isothermality (BIO2/BIO7 × 100) (6.6%), available nitrogen (3.9%), temperature seasonality (standard deviation × 100) (2.8%), precipitation seasonality (coefficient of variation) (2.2%), annual mean temperature (0.8%), total potassium (0.5%) and available phosphorus (0.4%) (Table 1).

*P. filicula* is sensitive to altitude and temperature, and climate is a dominating niche factor. Only three soil condition variables were in the top 10 indicators, and their contributions were low. The cumulative contribution rate of available nitrogen, total potassium and available phosphorus was only 4.8% of the total variation. These results showed that the surface soil conditions had a limited influence on the spatial distribution of *P. filicula* (Table S1).

### 2.3. Current Potential Distribution of P. filicula

**Figure 2:**
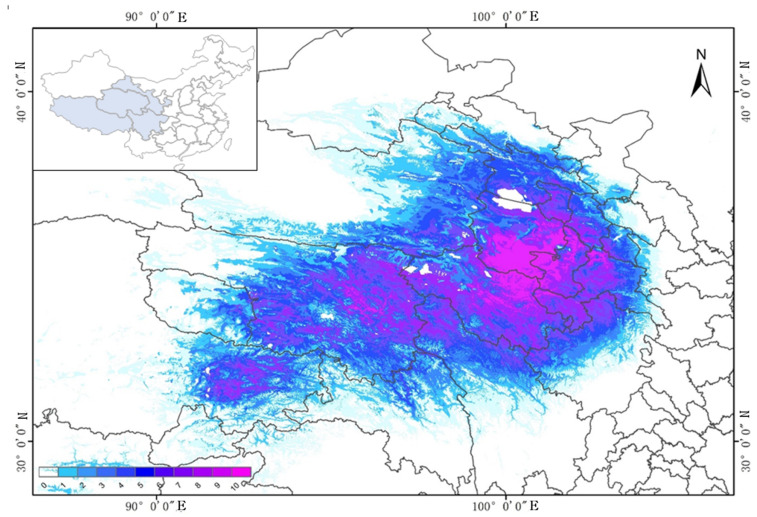
Current distribution of MaxEnt models for *Pomatosace filicula.* The icon 0–10 indicates that the higher the value, the more suitable the current habitat is for the growth of *Pomatosace filicula*.

The habitat distribution of *P. filicula* was divided into four levels according to suitability. The total suitable area was approximately 49.66 × 10^4^ km^2^. Figure 2 shows that *P. filicula* is distributed in the Qinghai, Sichuan, Tibet and Gansu provinces. The high-suitability area covered 1.74 × 10^4^ km^2^, accounting for 0.18% of China’s land area, and was mainly distributed near the Three-River Region in the southeast of Qinghai province. The medium-suitability area covered 20.38 × 10^4^ km^2^, accounting for 2.87% of China’s land area, and was mainly distributed in central and eastern Qinghai province, the Qinghai–Tibet junction area, the Qinghai–Sichuan junction area and the Gansu–Qinghai junction area. The low-suitability area covered 27.54 × 10^4^ km^2^, accounting for 5.17% of the China land area, and was mainly distributed in Qinghai, eastern Tibet, northwest Sichuan and southwest Gansu provinces.

### 2.4. Prediction and Fluctuation Analysis of Future Suitable Habitat Distribution

**Figure 3:**
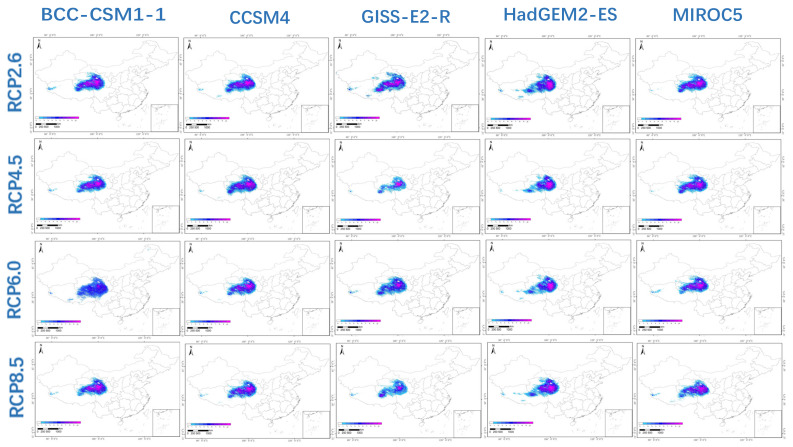
The habitat suitability of *Pomatosace filicula* population under four climate scenarios (RCP 2.6, RCP 4.5, RCP 6.0 and RCP 8.5) and five General Circulation models (BCC-CSM1-1, CCSM4, GIS-E2-R, HadGEM2-ES and MIROC) in 2050.

**Figure 4:**
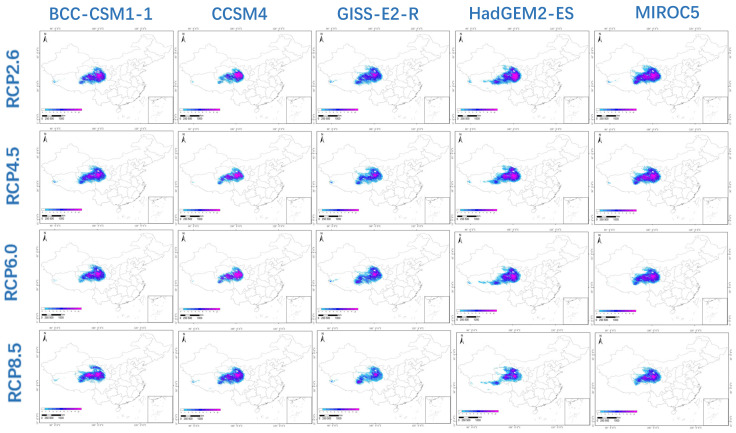
The habitat suitability of *Pomatosace filicula* population under four climate scenarios (RCP 2.6, RCP 4.5, RCP 6.0 and RCP 8.5) and five General Circulation models (BCC-CSM1-1, CCSM4, GIS-E2-R, HadGEM2-ES and MIROC5) in 2070.

The fifth IPCC report describes four future warming scenarios that form the basis for the total radiative forcing by 2100: RCP2.6, RCP4.5, RCP6.0 and RCP8.5. The five GCMs used in this study were BCC-CSM1-1, CCSM4, GIS-E2-R, HadGEM2-ES and MIROC5. To understand the impact of climate change on the habitat of *P. filicula* in China, we predicted the 2050 and 2070 *P. filicula* distribution patterns based on these 20 models. The distribution results are shown in Figure 3 and Figure 4.

**Figure 5:**
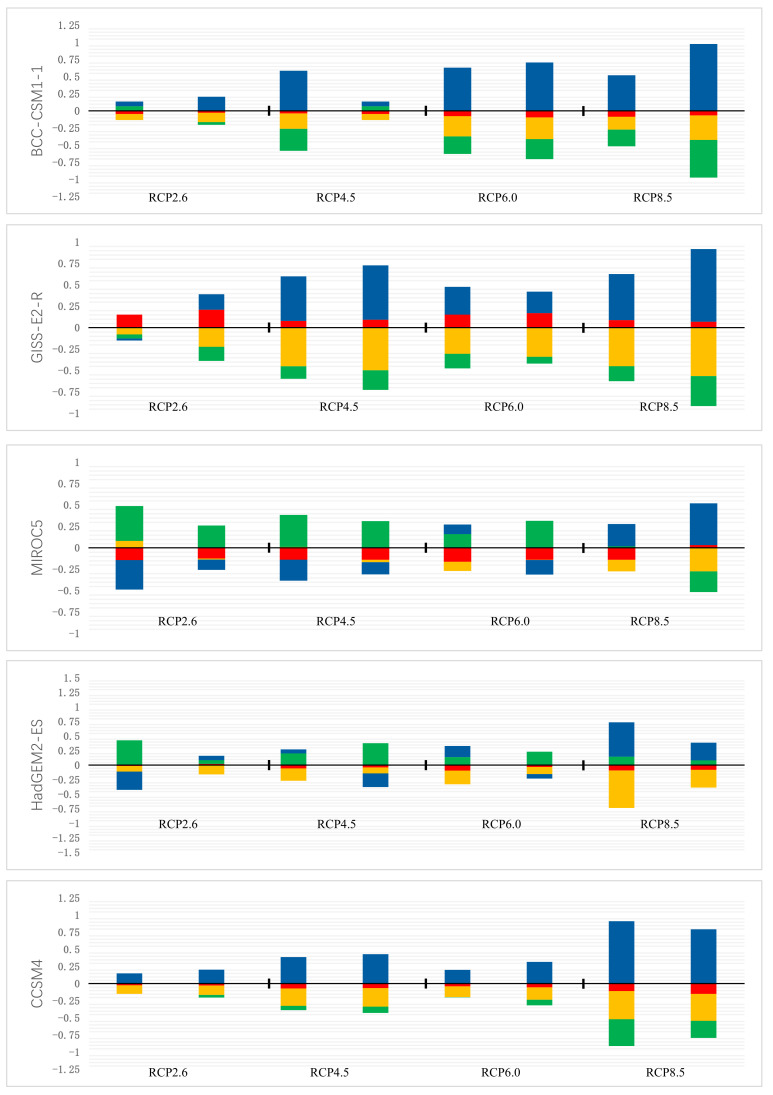
Four different levels of fluctuation in China’s future climate scenarios: Green, orange, red and blue represent the proportion of high, medium and low suitability areas and unsuitable habitats, respectively. A positive value indicates an increase in area and a negative value indicates a decrease in area.

RCP2.6 assumes good control of greenhouse gas concentrations in a minimum emissions scenario; thus, global warming was not significant in this model. Under this scenario, the future suitable area of *P. filicula* would increase in 2050 and then slightly shrink in 2070, with an average contraction of 2.7%, and the distribution pattern would not significantly change compared with today’s pattern. Under the RCP8.5 scenario, which assumes the highest greenhouse gas emissions, the unsuitable areas in 2050 and 2070 would expand by 54.65% and 68.20%, respectively, on average compared with the current area, and the suitable area in Gansu and Sichuan provinces would shrink. The RCP4.5 and RCP6.0 scenarios assume moderate greenhouse gas control. The changes in suitable area were similar under these two scenarios. Compared with the current area, for RCP4.5 and RCP6.0, the unsuitable area would expand by 26.34% and 26.17%, respectively, in 2050 and by 23.25% and 20.52%, respectively, in 2070 (Figure 5). Under these two greenhouse gas concentration paths, the total suitable area in 2070 would be larger than that in 2050. The detailed data are shown in Table S2.

## 3. Discussion

Generally, environmental variables are composed of abiotic environmental factors and biological environmental factors. Abiotic environmental factors mainly refer to climatic environmental variables, which simulate the distribution pattern and range of species in large-scale space [35]. In addition, large-scale hydrothermal conditions (including temperature and precipitation) are important factors in determining species distribution. Biological environmental factors include variables that are directly related to species distribution and usually act on species distribution on a small scale [29]. Their impact on species distribution is relatively complex. Furthermore, the role of biological factors may become insignificant at larger spatial scales due to weakened interactions between species [36]. Therefore, this study used common large-scale bioclimatic and soil variables to simulate the distribution range and pattern of *P. filicula* in the QTP.

### 3.1. Analysis of Key Environmental Variables

The potential distribution of *P. filicula* in China was obtained with ArcGIS software and the MaxEnt model, using field survey and distribution data obtained from the literature. In this study, the relationships between key environmental variables and the occurrence probability of *P. filicula* were analyzed, and the corresponding response curves were obtained. The results showed that the occurrence probability of *P. filicula* changed with changes in key environmental variables. The most important factor affecting the distribution of *P. filicula* was predicted to be altitude (alt 52.4%), and its optimum altitude was 4000–4500 m (Figure S3), which is consistent with previous studies and sampling results.

Annual precipitation (BIO12 15.3%) affects the growth and distribution of *P. filicula*, and the annual precipitation suitable for the survival of *P. filicula* is 400–500 mm (Figure S3). In addition, the results showed that 4 of the top 10 key factors were related to temperature: temperature annual range (BIO7 15.1%), isothermality (BIO3 6.6%), temperature seasonality (BIO4 2.8%) and annual mean temperature (BIO1 0.8%) and climate (temperature and precipitation) contributed 42.8%; therefore, climate change should be considered as a second key factor affecting the distribution of *P. filicula* in addition to altitude.

High altitudes will be directly affected by climate change within a certain range. As the air becomes cold and dry, it indirectly affects the growth of *P. filicula* [27,28,29]. In addition, growing at high altitude is a typical feature of apple as an alpine species Temperature and precipitation directly affect plant respiration and transpiration, and have a direct effect on plant growth and development [23]. Large area hydrothermal conditions (including temperature and precipitation) are important factors determining species distribution [11,23]. This explains why altitude and climate factors have a greater impact than soil factors on species distribution. According to the prediction, *P. filicula* can grow at high altitudes with low temperatures and humid conditions.

### 3.2. Potential Environmental Change Trends in the Qinghai–Tibet Plateau

Current research shows that climate change will result in global temperatures continuing to rise [37], likely reaching temperatures 1.5 °C or higher than pre-industrialization temperatures [5]. The QTP has experienced a significant warming process, and the warming rate is higher than the global average. From 1961 to 2020, the annual average temperature of the QTP increased by 0.35 °C every 10 years—more than twice the global warming rate in the same period [35,38]. The China Meteorological Administration has found that the future warming trend of the QTP is positively correlated with the intensity of external radiative forcing. The warming of the plateau under the high-CO_2_ emission scenario would be more intense than that under the medium- and low-CO_2_ scenarios. Warming will probably exceed 6.5 °C by the end of the 21st century, and the entire QTP will experience significant warming in the next few decades. Although there are considerable uncertainties in these scenarios, the simulated 2050 and 2070 climate conditions indicate that the entire QTP will experience significant warming over the next few decades. Combined with future precipitation trends, Yw, Liang, Jw, Ww, Nn, Yg and Xw predict that the Tibetan Plateau will become warmer and drier [25].

**Figure 6:**
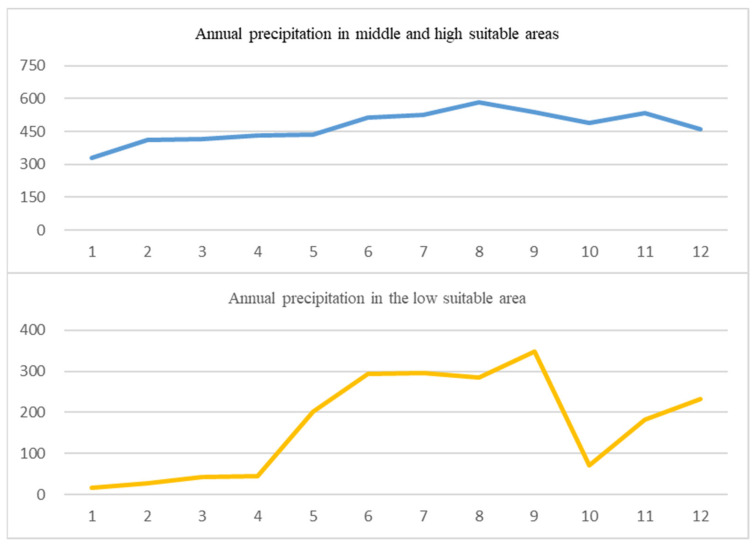
Precipitation of 24 observation points in the middle- and high-suitability area and low-suitability area of the Qinghai–Tibet Plateau from 1952 to 2016. Blue represents the annual precipitation in medium- and high-suitability areas, Orange represents the annual precipitation in low-suitability areas.

From the meteorological data center of the China Meteorological Administration (www.cma.gov.cn/en2014/, accessed on 9 October 2021), we collected the annual precipitation values of 12 observation points distributed throughout the medium-, high- (near the Three-River Region) and low-suitability *P. filicula* habitat areas of the QTP. In the medium- and high-suitability areas with concentrated distributions of *P. filicula*, the average annual precipitation was 472 mm. By comparison, the average annual precipitation in the surrounding low-suitability area was much lower at 170 mm (Figure 6). Specific data are shown in Table S3. Jiang, Zhang, Gao, Cai, Zhou, Li and Zhang [10] found that compared with current levels, the annual average precipitation in the Three-River Region will increase slightly by 0–30 mm in 2050 and by 0–50 mm in 2070. This increased precipitation may be why the distribution range of *P. filicula* is expected to shrink and become concentrated in the Three-River Region areas under future climate warming scenarios.

### 3.3. Response Measures and Problems

*P. filicula* is an endemic species in China and is listed as a national secondary protected plant with important medicinal and conservation significance [33]. It is an alpine crop distributed on the QTP and has regional characteristics due to long-term adaptation to the plateau environment [39]. Because alpine plants are distributed over a limited range, they are more sensitive to climate change because of their limited capacities for seed dispersal and long-distance migration [40,41]. Compared with temperate plants, many alpine plants have a smaller migration range and are more vulnerable to extinction in the face of global warming [42]. As the QTP becomes warmer and drier in the future [21,22], the area in which *P. filicula* can grow (cold and humid conditions) will decrease, posing a great threat to the species.

**Figure 7:**
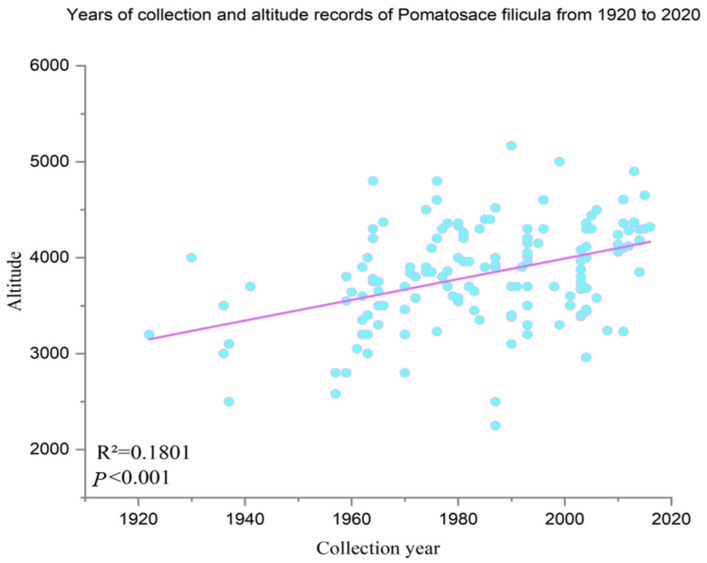
Statistical year and elevation of *P. filicula* collected in 1920–2020. The blue dots represent the year in which the *P. filicula* were collected and the corresponding elevation; the blue dashed line represents the trend line of the elevation at which *P. filicula* was collected with increasing years.

*P. filicula* is reproduction method is seed dispersal, which is more capable of migration than asexual reproduction to a certain extent. In order to verify whether *P. filicula* has the potential to migrate to suitable habitats, we collected 195 acquisition points from 1920 to 2020, and the results are shown in Figure 7. As the year increased, the acclimation zone height of *P. filicula* was also increasing, and the elevation trend line moves upward. Our experiments are meaningful and protected areas should be set up in suitable areas in the future. Therefore, we believe that *P. filicula* have a certain ability to avoid hazards and can migrate to areas suitable for growth in the future. Our experiment is meaningful. We should take protective measures and further establish protected areas in the future suitable areas.

At present, many different grades and types of national parks, national nature reserves and provincial nature reserves have been established in the QTP. However, most of these areas were established to protect wild animals, desert ecosystems, wetlands and endangered wildlife [43]. Therefore, we should strengthen protections for species diversity in the QTP. Endangered plants should be added as worthy of protection to deepen the justification for these established protected areas. Finally, effective protection plans and preventative measures should be adopted. Regions with low- and medium-suitability habitats should actively engage in educating the public about the importance of protecting endangered plants. When *P. filicula* is encountered, it should not be excavated; rather, local protective measures should be taken. In provinces with highly suitable areas, more attention should be given to protecting the current regional environment.

In this study, the MaxEnt model in ArcGIS was used to quantitatively and intuitively analyze the current and future spatial distribution patterns of *P. filicula* in China and the factors affecting these patterns. To improve the accuracy of these predictions, future work should consider uncontrollable human factors and other variables such as ultraviolet-B radiation.

## 4. Materials and Methods

### 4.1. Data and Variable Sources

Data were obtained from the following sources: CNKI database (http://www.cnki.net/, accessed on 7 July 2021), China Digital Herbarium (https://www.cvh.ac.cn/, accessed on 7 July 2021), NSII China National Specimen Resource Platform (http://www.nsii.org.cn/2017/home.php, accessed on 7 July 2021), Global Biodiversity Information Facility (https://www.gbif.org/zh/, accessed on 7 July 2021) and China Plant Image Library (http://ppbc.iplant.cn/, accessed on 7 July 2021). When a record was missing precise geographic coordinates, we used Google Earth (http://ditu.google.cn/, accessed on 7 July 2021) to determine the latitude and longitude based on the geographic location described. The latitude and longitude coordinates of the sample were stored in Excel and converted to CSV format to build the MaxEnt model.

In total, 19 bioclimatic and altitude variables were downloaded from the WorldClim Global Climate Database (version 1.4) (http://www.worldclim.org/, accessed on 7 July 2021) at a spatial resolution of 30 arc-seconds (approximately 1 km). These variables are the most widely used climatic variables in species distribution models. Eight soil variables were downloaded from the National Tibetan Plateau Data Center (https://data.tpdc.ac.cn/zh-hans/, accessed on 7 July 2021). We resampled the 8 soil variables at a spatial resolution of 30 arc-seconds. Specific environmental factors are shown in Table S2. The base map of China was obtained from the National Basic Geographic Information System (http://nfgis.nsdi.gov.cn/, accessed on 7 July 2021). We sampled the geographic variables at a spatial resolution of 30 arc-seconds.

Future bioclimatic variables for 2050 and 2070 were taken from four Intergovernmental Panel on Climate Change (IPCC)-CMIP5 representative concentration pathways (RCPs): RCP2.6, RCP4.5, RCP6.0 and RCP8.5. These RCPs represent the full range of possible values for the total radiative forcings of +2.6, +4.5, +6.0 and +8.5 W/m^2^, respectively. BCC-CSM1–1, CCSM4, GISS-E2-R, HadGEM2-ES and MIROC5 are different global climate models (GCMs). The climate scenario data were obtained from the International Center for Tropical Agriculture (http://ccafs-climate.org/, accessed on 7 July 2021). All bioclimatic variables were converted to ASCII format for MaxEnt analysis.

### 4.2. Environmental Variable Processing

Previous studies have revealed serious problems of multicollinearity among bioclimatic variables [44,45]. To select variables for the model with strong predictive power, we eliminated the multiple linear relationships between variables and established Pearson correlation coefficients for the 28 environmental variables in 107 species occurrence records [46]. The EMTools program was used to calculate the Pearson correlation coefficients between the 28 variables using data related to Chinese territory. A threshold of 0.8 was used to indicate a significant correlation [47]; only environmental variables with a correlation coefficient of less than 0.8 were used to construct the MaxEnt model [48,49]. Contribution analysis of environmental factors with a correlation less than 0.8 was carried out with Maxent, and environmental factors with a low contribution or 0 were excluded [48].

### 4.3. Species Distribution Model Evaluation

MaxEnt is a density estimation and species distribution prediction model based on maximum entropy theory [50]. By comparing the true skill statistics, we found that the value of MaxEnt was the closest to 1, indicating that this model had the best accuracy [51]. In the absence of species distribution data, MaxEnt still performed well in generating accurate predictions [52,53,54]. This model has been widely used in conservation biology, invasion biology and other fields [49,54,55,56,57,58].

#### 4.3.1. MaxEnt Parameter Optimization

The MaxEnt was used to predict the potential distribution of MaxEnt in the QTP. We randomly selected 75% of occurrence records as training data for model building and the remaining 25% as testing data for model evaluation. Jackknife was used to evaluate variable contributions, and the options of ‘create response curves’ were selected. The model has been repeated 10 times and the maximum iterations as 5000 [59]. We used the “10 percentile training presence logistic threshold” to define the suitable and unsuitable habitats for species distribution [49]. Other parameters used default settings according to the MaxEnt model. The area under the receiving operator characteristic curve (AUC) and the average omission error were used to assess the accuracy of each model prediction [60,61]. We used the grid cells to calculate the suitable and unsuitable habitat area of *P. filicula*. The finished product data from 1960–1990 and 2050 were in ASCII (.ASC) format. R model was used to run the script, and the parameter setting area was 73.1–135.5° E and 17.7–53.9° N. 28 environmental factors from finished product data (1960–1990) were projected to 2050 and 2070.

A habitat suitability curve for each variable was calculated, and the contribution of each variable to the *P. filicula* habitat model was calculated using the jackknife test. The AUC was used as an index to assess the accuracy of the suitability analysis results for *P. filicula*. The ROC curve is an acceptance curve in which the abscissa represents the false positive rate (1−specificity) and the vertical axis represents the true positive rate (1−omission rate) [60]. The model performance was classified as failed (0.5–0.6), poor (0.6–0.7), average (0.7–0.8), good (0.8–0.9) or excellent (0.9–1.0). An AUC value closer to 1 indicates a better model performance [37].

#### 4.3.2. Classification of Suitable Area

In total, 11 threshold statistics were obtained according to the MaxEnt results generated for 10 repeats. The habitat suitability index (0–1) for potential species distribution was calculated whereby a higher value indicated a higher fitness; 0 indicated a completely unsuitable habitat. The minimum training presence was used to divide the fitness index into four levels: unsuitable (0–0.3025), low suitability (0.3025–0.5350), medium suitability (0.5350–0.7675) and high suitability (0.7675–1.0).

#### 4.3.3. Parameter Optimization

Based on the above optimized parameter combination, a contemporary niche model was built with MaxEnt software and projected to the years 2050 and 2070. The model included 28 sets of environmental factor data and took into account 5 GCMs (BCC-CSM1-1, CCSM4, GISS-E2-R, HadGEM2-ES and MIROC5) and 4 RCPs (the minimum greenhouse gas emission scenario (RCP2.6), moderate greenhouse gas emission scenario (RCP4.5), high greenhouse gas emission scenario (RCP6.0) and maximum greenhouse gas emission scenario (RCP8.5)).

## 5. Conclusions

In this study, we simulated the current spatial distribution of *P. filicula*, tested the key niche indicators that affect its distribution and simulated its suitable habitat distribution in 2050 and 2070 under future climate change scenarios (four RCP scenarios and five global climate models). Our results show that:

(1) The current suitable habitat for *P. filicula* is located in the QTP and is mainly distributed in the southeast of Qinghai province. Suitable habitat is also scattered in northwestern Sichuan, Tibet and Gansu provinces.

(2) Elevation is the most important factor affecting the distribution of *P. filicula*, followed by temperature and precipitation. *P. filicula* is more suitable for growing in high-altitude areas that are humid and cold.

(3) As the temperature increases, the area of *P. filicula* habitat decreases. Under RCP8.5, *P. filicula* would lose more than half of its current habitat area. Under RCP6.0 and RCP4.5, *P. filicula* would lose a quarter of its current habitat. Under RCP2.6, the *P. filicula* habitat would increase by 2050 but decrease by 2.7% by 2070.

(4) Future global climate change will promote the migration of this species to higher altitudes that are more humid and colder. In the future, the suitable area will shrink to southeast Qinghai province and will mainly be distributed in the areas of the Three-River Region. Future warming and drying of the QTP will aggravate the deterioration of *P. filicula* habitat and increase the species’ survival risk.

(5) Based on these findings, in addition to controlling greenhouse gas emissions, the Ecological and environmental protection department should further strengthen nature reserves to protect the existing and future habitats of *P. filicula*.

## Figures and Tables

**Table 1:** The contribution proportions and cumulative proportion contributions of the top 10 indicators in principle component analysis (PCA).

Bioclimatic Variables	Description	Units	Contribution Percentages	Rankings
**alt**	Elevation	m	52.4	1
**bio12**	Annual precipitation	mm	15.3	2
**bio7**	Temperature annual range (BIO5–BIO6)	°C	15.1	3
**bio3**	Isothermality (BIO2/BIO7 × 100)	%	6.6	4
**an**	Available nitrogen	mg/kg	3.9	5
**bio4**	Temperature seasonality (standard deviation × 100)	%	2.8	6
**bio15**	Precipitation seasonality (coefficient of variation)	1	2.2	7
**bio1**	Annual mean temperature	°C	0.8	8
**tk**	Total potassium	mg/L	0.5	9
**ap**	Available phosphorus	mg/kg	0.4	10

## Data Availability

Not applicable.

## Supplementary Materials

The following supporting information can be downloaded at: https://www.mdpi.com/article/10.3390/plants11050670/s1. Table S1: Environmental indicators used in this paper to model the potential distribution of *Pomatosace filicula*. There are 8 soil and 1terrain indicators, and 19 bioclimatic indicators; Figure S1: In the current situation, MaxEnt model is used to analyze the receiver operating characteristic (ROC) curve and average test AUC of *Pomatosace filicula*; Figure S2: The results of the jackknife test of variables’ contribution in modelling *Pomatosace filicula* distribution in the current situation; Table S2 Comparison between the current distribution and the suitable areas of *P. pinnatifida* in 2050 and 2070 under four climate scenarios; Figure S3: Response curves of 10 environmental variables in habitat distribution model of *Pomatosace filicula*; Table S3: Precipitation of 24 observation points in the middle and high suitable area and low suitable area of the Qinghai Tibet Plateau from 1952 to 2016; Figure S4: In the current situation, MaxEnt model is used to analyze the receiver operating characteristic (ROC) curve and average test AUC of *Pomatosace filicula*; Figure S5: The results of the jackknife test of variables’ contribution in modelling *Pomatosace filicula* distribution in 2050 and 2070 under four climate scenarios and five general circulation models.
