# Supplementary material for: MaxEnt Modeling to Predict the Current and Future Distribution of Pomatosace filicula under Climate Change Scenarios on the Qinghai–Tibet Plateau
Source: Plants (Basel). 2022 Feb 28;11(5):670. doi: 10.3390/plants11050670 (PMC8912362; doi:10.3390/plants11050670)

Supplemental data

Article title: MaxEnt Modeling to Predict the Current and Future Distribu-tion of *Pomatosace filicula* under Climate Change Scenarios on the Qinghai– Tibet Plateau

Authors: Kaiyang Chen, Bo Wang, Chen Chen and Guoying Zhou *

Table. S1 Environmental indicators used in this paper to model the potential distribution of *Pomatosace filicula*. There are 8 soil and 1terrain indicators, and 19 bioclimatic indicators

Bioclimatic variables	Description	Unit
Bio1	Annual mean temperature	° C
Bio2	Mean diurnal range	° C
Bio3	Isothermality (BIO2/BIO7) (* 100)	%
Bio4	Temperature seasonality (standard deviation *100)	° C
Bio5	Max temperature of warmest month	° C
Bio6	Min temperature of coldest month	° C
Bio7	Temperature annual range (BIO5-BIO6)	° C
Bio8	Mean temperature of wettest quarter	° C
Bio9	Mean temperature of driest quarter	° C
Bio10	Mean temperature of warmest quarter	° C
Bio11	Mean temperature of coldest quarter	° C
Bio12	Annual precipitation	mm
Bio13	Precipitation of wettest month	mm
Bio14	Precipitation of driest month	mm
Bio15	Precipitation seasonality (coefficient of variation)	1
Bio16	Precipitation of wettest quarter	mm
Bio17	Precipitation of driest quarter	mm
Bio18	Precipitation of warmest quarter	mm
Bio19	Precipitation of coldest quarter	mm
alt	Elevation	m
ph	Topsoil pH (H2O)	log(H+)
an	Available nitrogen	mg/kg
ak	Available potassium	mg/kg
ap	Available phosphorus	mg/kg
tn	Total nitrogen	mg/L
tk	Total potassium	mg/L
tp	Total phosphorus	mg/L
Som	soil organic matter	g/kg

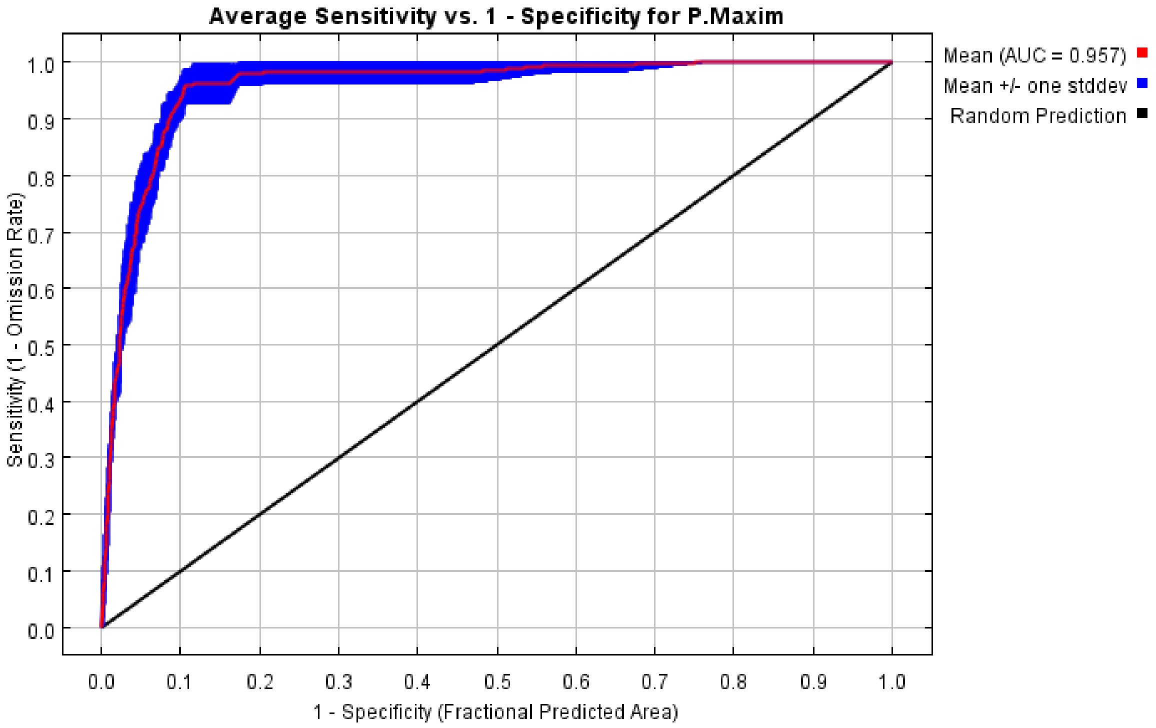

Figure S1 In the current situation, MaxEnt model is used to analyze the receiver operating characteristic (ROC) curve and average test AUC of *Pomatosace filicula*.

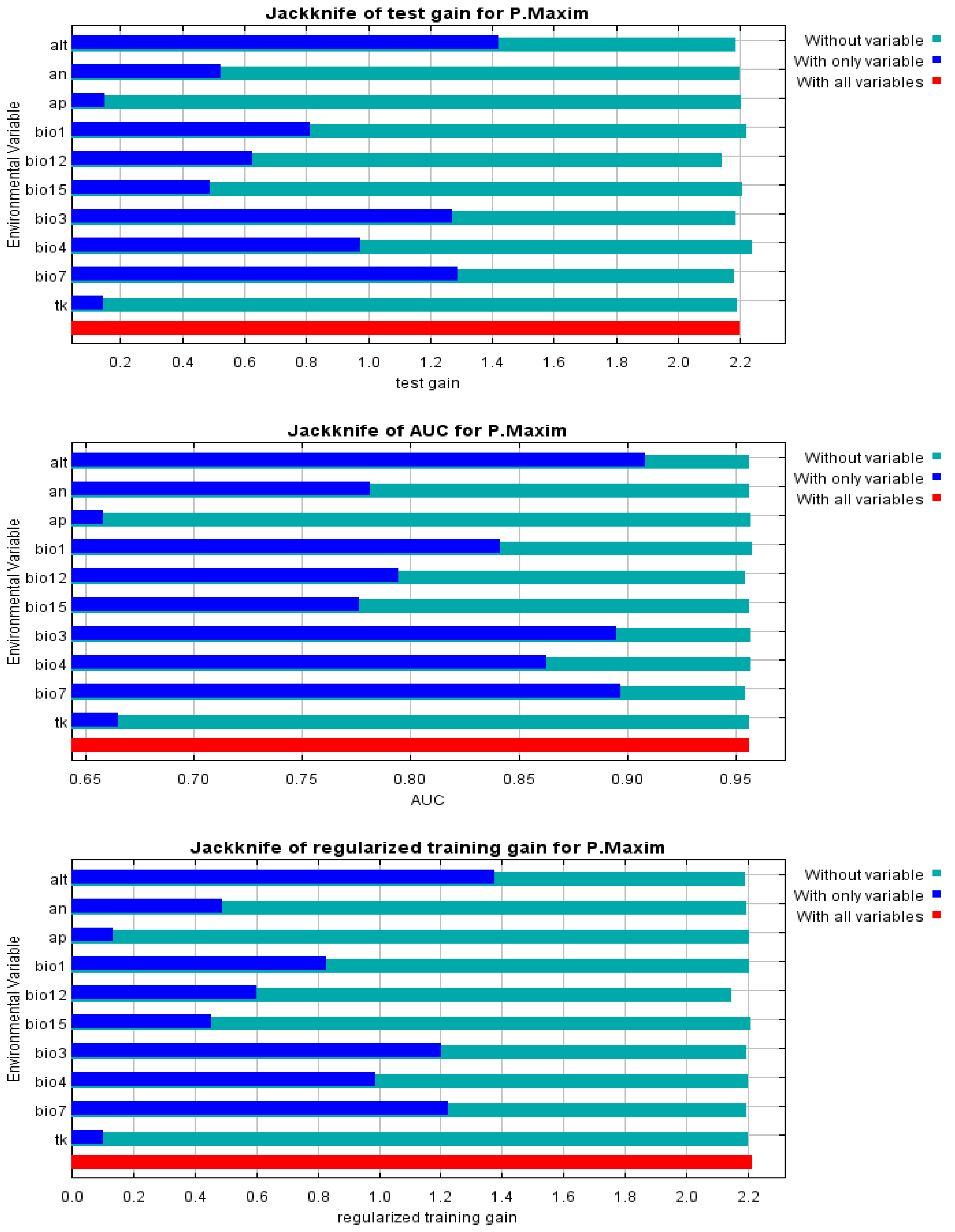

Figure S2 The results of the jackknife test of variables' contribution in modelling *Pomatosace filicula* distribution in the current situation (The regularized training gain describes how much better the Maxent distribution fits the presence data compared to a uniform distribution. The dark blue bars indicate that the gain from using each variable in isolation, the light blue bars indicate the gain lost by removing the single variable from the full model, and the red bar indicates the gain using all of the variables).

Table. S2 Comparison between the current distribution and the suitable areas of P. pinnatifida in 2050 and 2070 under four climate scenarios (RCP 2.6, RCP 4.5, RCP 6.0 and RCP 8.5) and five general circulation models (BCC-CSM1-1、

			**Total suitable region**	**Lowly suitable region**	**Moderately suitable region**	**Highly suitable region**	**unsuitable habitat**
GISS-E2-R	**RCP2.6**	**2050**	2.30%	15.08%	-7.87%	-4.91%	-2.30%
		**2070**	2.03%	20.85%	-22.43%	-16.52%	18.10%
	**RCP4.5**	**2050**	-14.54%	7.73%	-45.14%	-14.66%	52.07%
		**2070**	-15.99%	9.16%	-49.88%	-22.81%	63.53%
	**RCP6.0**	**2050**	-4.55%	15.18%	-30.72%	-16.98%	32.52%
		**2070**	-4.64%	16.92%	-34.05%	-8.04%	25.16%
	**RCP8.5**	**2050**	-14.00%	8.80%	-45.02%	-17.59%	53.81%
		**2070**	-20.50%	6.86%	-56.71%	-34.93%	84.78%
CCSM4	**RCP2.6**	**2050**	-6.22%	-2.47%	-12.41%	0.20%	14.68%
		**2070**	-7.11%	-2.84%	-13.72%	-3.89%	20.45%
	**RCP4.5**	**2050**	-14.38%	-7.14%	-25.38%	-6.24%	38.76%
		**2070**	-14.87%	-6.55%	-27.10%	-9.19%	42.84%
	**RCP6.0**	**2050**	-8.43%	-4.01%	-15.64%	-0.45%	20.10%
		**2070**	-10.54%	-5.43%	-18.19%	-8.15%	31.77%
	**RCP8.5**	**2050**	-24.13%	-10.90%	-41.20%	-38.99%	91.08%
		**2070**	-25.17%	-15.00%	-39.38%	-24.87%	79.25%
HadGEM2-ES	**RCP2.6**	**2050**	-3.05%	-1.43%	-9.74%	42.58%	-31.41%
		**2070**	-4.96%	2.01%	-15.93%	6.56%	7.36%
	**RCP4.5**	**2050**	-10.92%	-5.58%	-21.33%	20.14%	6.78%
		**2070**	-4.85%	-4.03%	-10.19%	37.70%	-23.48%
	**RCP6.0**	**2050**	-14.18%	-9.39%	-23.57%	13.85%	19.11%
		**2070**	-5.71%	-3.21%	-12.11%	23.05%	-7.73%
	**RCP8.5**	**2050**	-32.53%	-9.20%	-64.28%	14.50%	58.98%
		**2070**	-16.53%	-7.93%	-30.75%	8.07%	30.62%
BCC-CSM1-1	**RCP2.6**	**2050**	-5.73%	-4.80%	-8.63%	6.64%	6.78%
		**2070**	-7.11%	-2.84%	-13.72%	-3.89%	20.45%
	**RCP4.5**	**2050**	-12.26%	-3.84%	-22.46%	-32.21%	58.50%
		**2070**	-5.73%	-4.80%	-8.63%	6.64%	6.78%
	**RCP6.0**	**2050**	-17.20%	-7.88%	-29.58%	-25.63%	63.09%
		**2070**	-19.18%	-9.76%	-31.51%	-29.30%	70.58%
	**RCP8.5**	**2050**	-13.16%	-8.59%	-18.89%	-24.48%	51.96%
		**2070**	-20.19%	-6.77%	-35.81%	-55.02%	97.60%
MIROC5	**RCP2.6**	**2050**	-2.97%	-14.32%	8.03%	40.79%	-34.50%
		**2070**	-6.33%	-12.55%	-1.25%	25.90%	-12.10%
	**RCP4.5**	**2050**	-5.80%	-13.96%	0.95%	37.44%	-24.42%
		**2070**	-7.65%	-14.02%	-2.90%	31.12%	-14.19%
	**RCP6.0**	**2050**	-12.72%	-16.37%	-10.77%	15.95%	11.18%
		**2070**	-6.52%	-13.99%	-0.23%	31.41%	-17.19%
	**RCP8.5**	**2050**	-13.12%	-13.99%	-13.72%	1.69%	26.02%
		**2070**	-10.25%	3.12%	-27.66%	-24.20%	48.75%

CCSM4、GIS-E2-R、HadGEM2-ES and MIROC

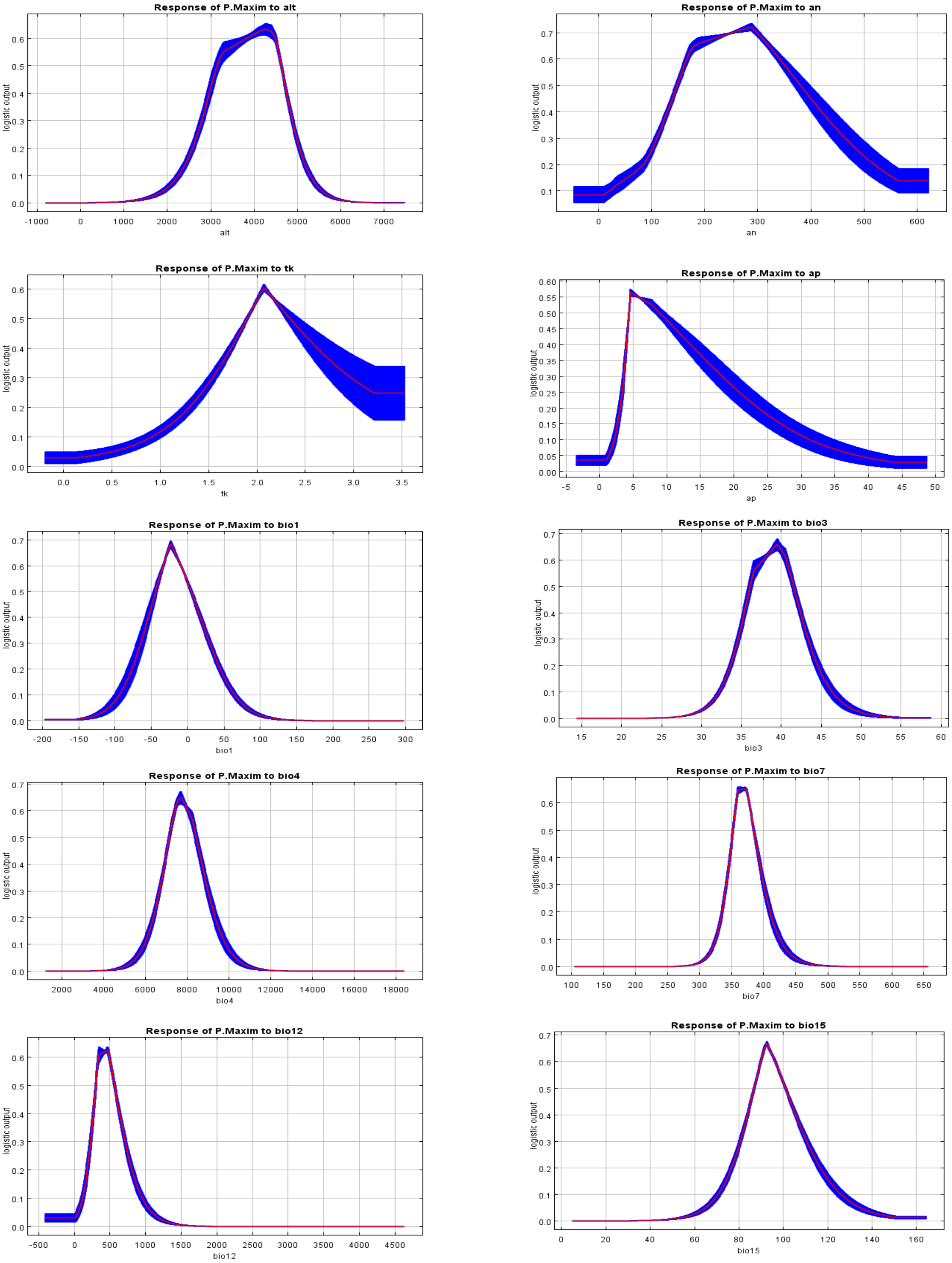

Figure S3 Response curves of 10 environmental variables in habitat distribution model of *Pomatosace filicula*. alt: elevation(m), an Available :nitrogen(mg/kg), tk: Total potassium(mg/L),ap: Available phosphorus(mg/kg), bio1:Annual mean temperature(°C), bio3: Iso-thermality (BIO2/BIO7× 100)(%),bio4:Temperature seasonality (standard deviation ×100)(%), bio7:Temperature annual range (BIO5-

Table. S3 Precipitation of 24 observation points in the middle and high suitable area and low suitable area of the Qinghai Tibet Plateau from 1952 to 2016.

medium and high suitable areas	Annual precipitation	low suitable area	Annual precipitation
Ping’an	329.7	Lenghu	16.4
Qumalai	412.4	Xiaozaohuo	27.9
Quinan	416.3	Geermu	42.6
Tongde	430.9	Nuomuhong	45.4
Naqu	434.8	Dulan	202.9
Qinghaihu	513.2	Wudaoliang	293.8
Zaduo	527.3	Tuotuohe	294.7
Henan	583.8	Jiangzi	284.7
Nangqian	536.4	Minhe	347.0
Yushu	488.4	Shiquanhe	71.4
Huangzhong	533.2	Gaize	181.5
Hualong	460.5	Jingyuan	232.5

plants MDPI

Figure S4 In the current situation, MaxEnt model is used to analyze the receiver operating characteristic (ROC) curve and average test AUC of *Pomatosace filicula*. Four climate scenarios (RCP 2.6, RCP 4.5, RCP 6.0 and RCP 8.5) and five general circulation models (BCC-CSM1-1C-CSM1-1、CCSM4、GIS-E2-R、HadDGEM2-ES and MIROC5) in 2050 and 2070 respectively.

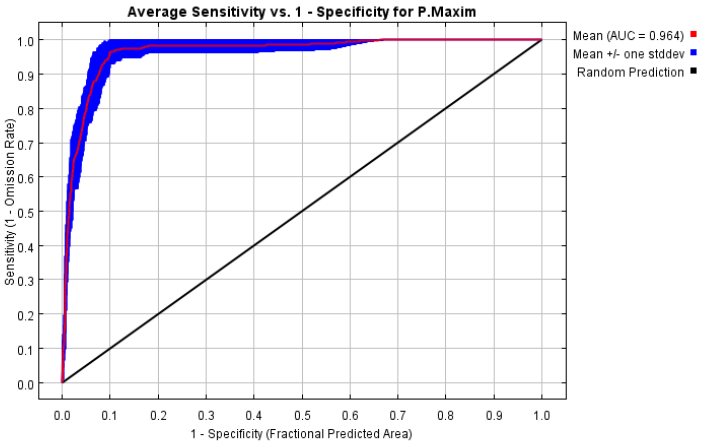

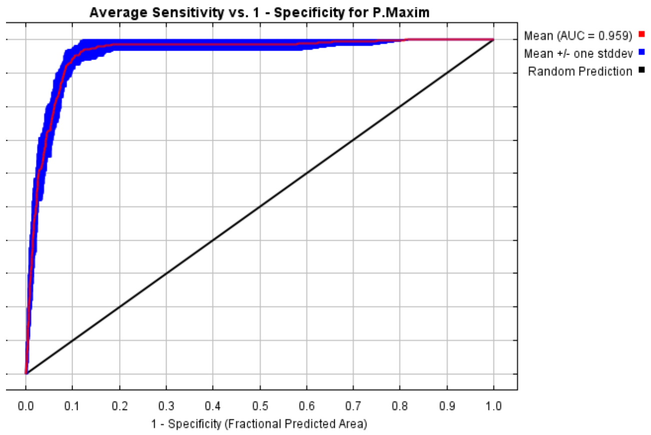

MIROC5-RCP2.6-2050 **MIROC5-RCP2.6-2070**

MIROC5-RCP4.5-2050

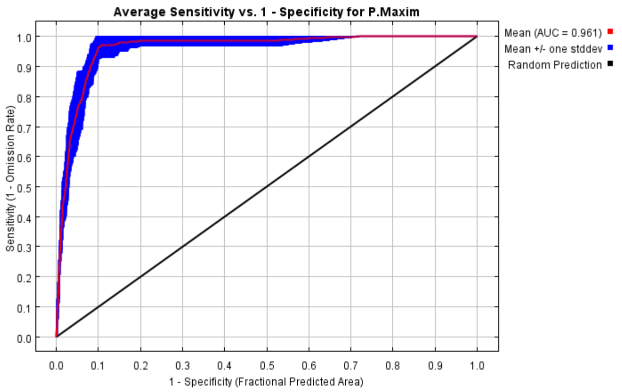

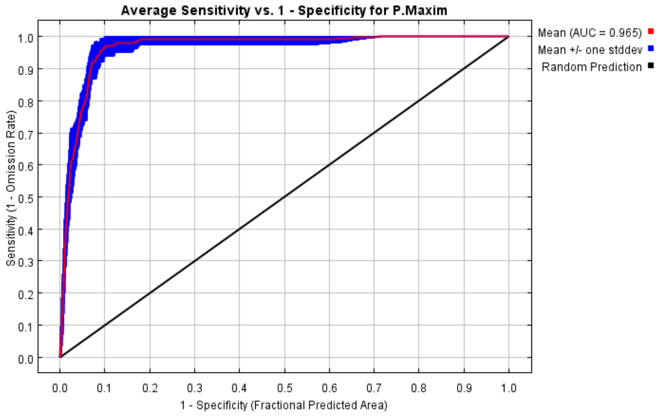

MIROC5-RCP4.5-2070

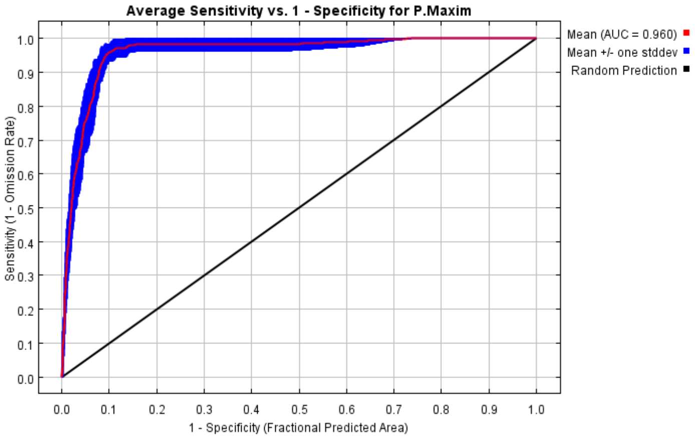

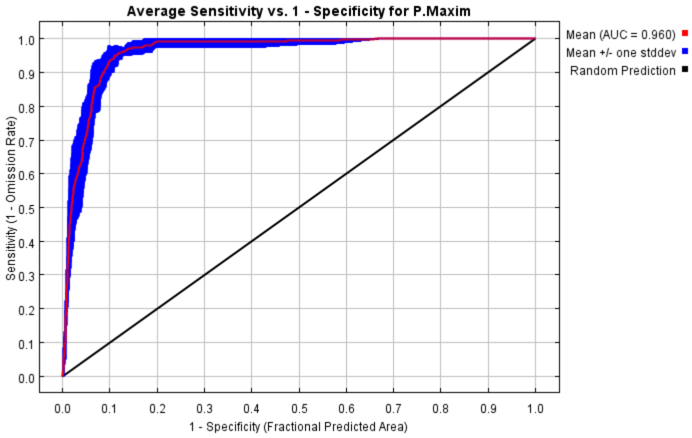

MIROC5-RCP6.0-2050 **MIROC5-RCP6.0-2070**

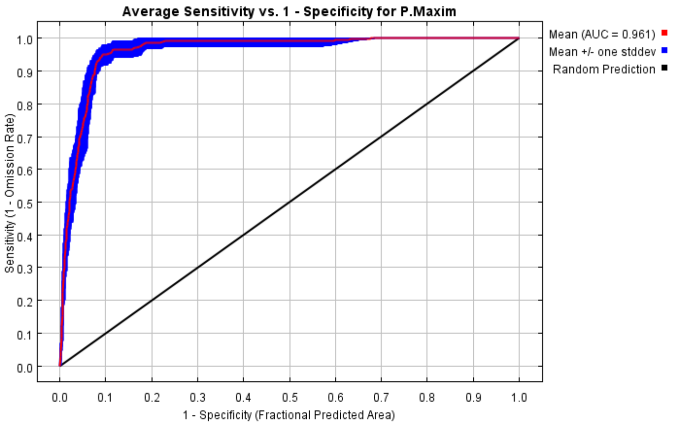

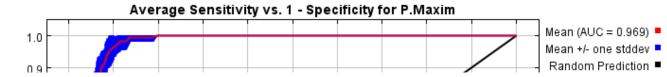

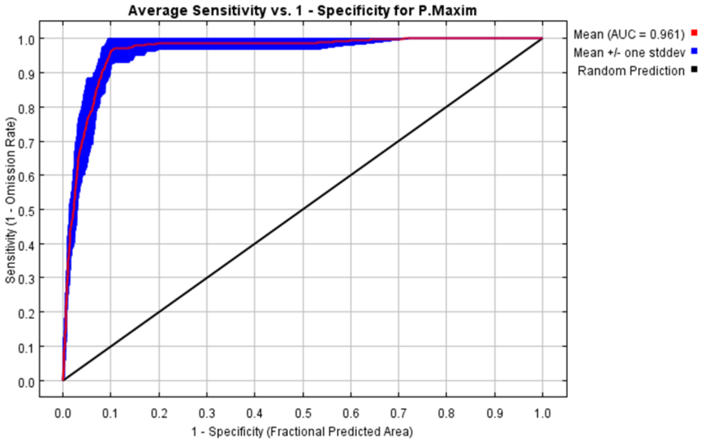

MIROC5-RCP6.0-2050

MIROC5-RCP8.5-2070

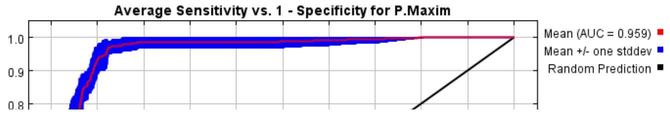

BCC-CSM1-1-RCP2.6-2050 **BCC-CSM1 -1-RCP2.6-2050**

BCC-CSM1-1-RCP4.5-2050 **BCC-CSM1-1-RCP4.5-2070**

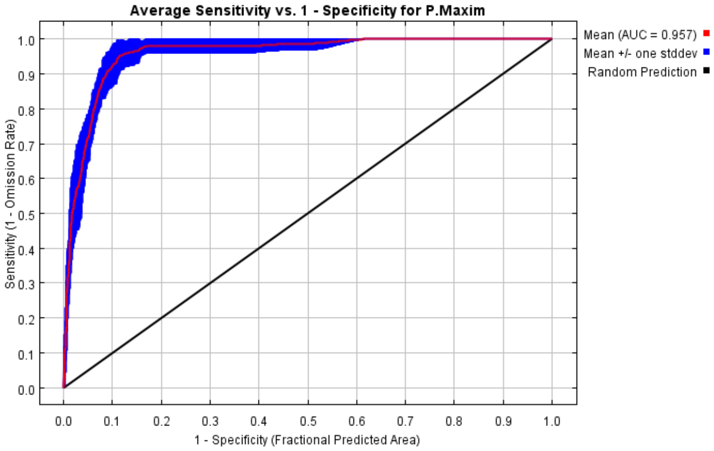

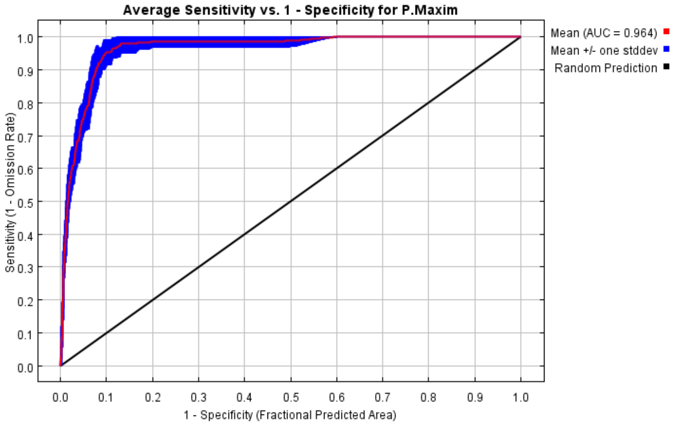

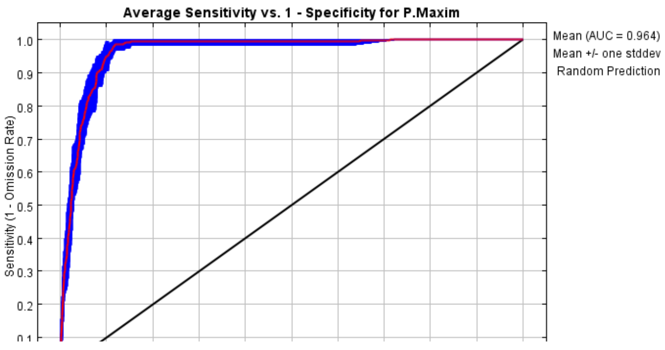

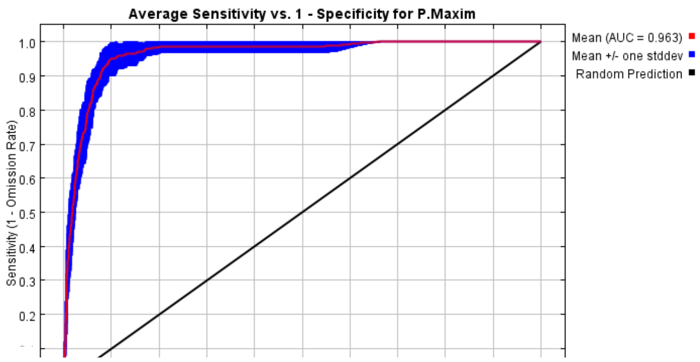

BCC-CSM1-1-RCP6.0-2050

BCC-CSM1-1-RCP6.0-2070

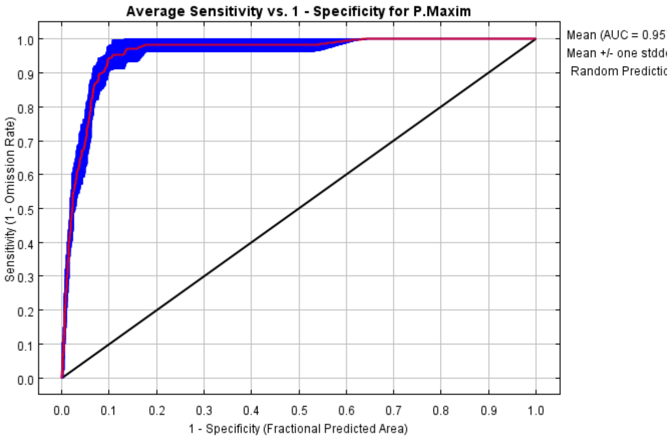

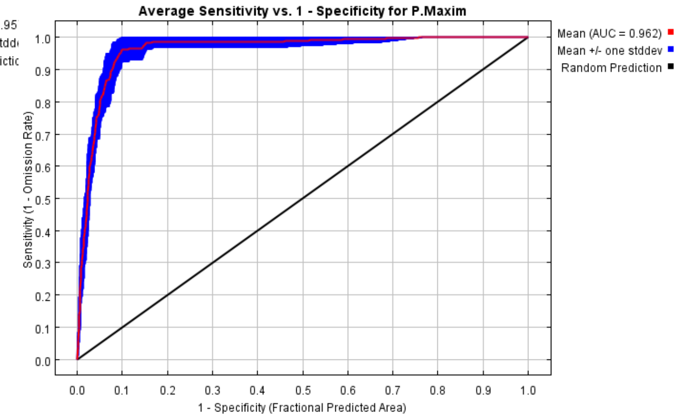

BCC-CSM1-1-RCP8.5-2050

BCC-CSM1-1-RCP8.5-2070

CCSM4-RCP2.6-2050

CCSM4-RCP2.6-2070

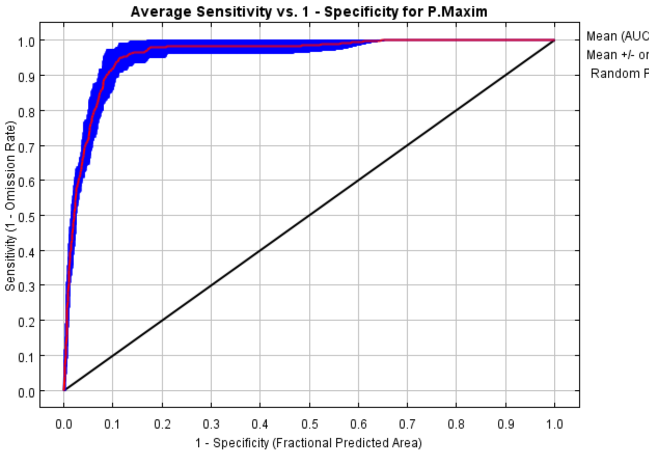

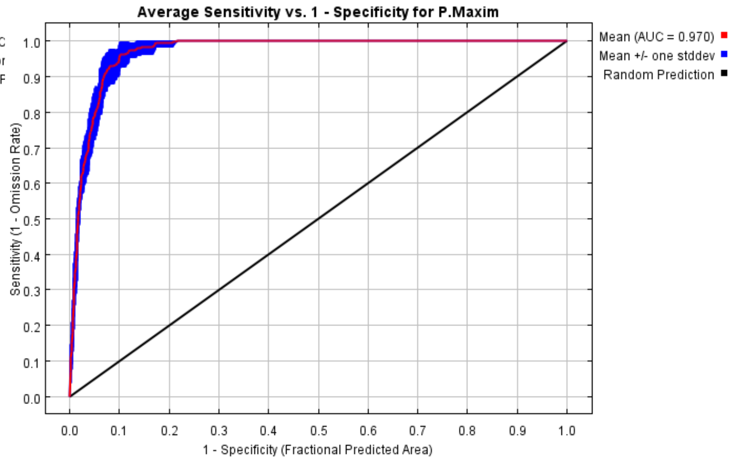

CCSM4-RCP4.5-2050

CCSM4-RCP4.5-2070

CCSM4-RCP6.0-2050

CCSM4-RCP6.0-2070

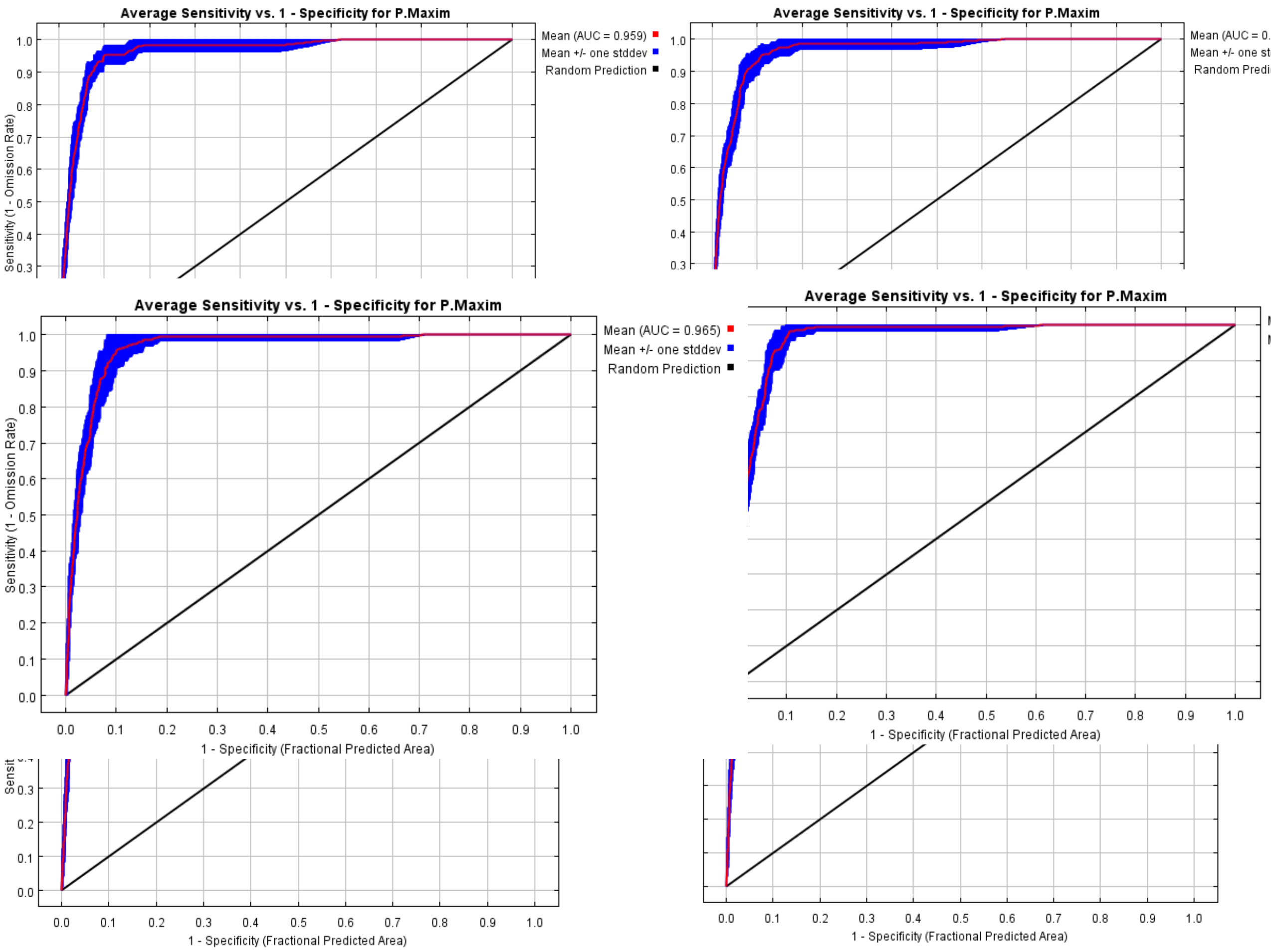

CCSM4-RCP8.5-2050 CCSM4-RCP8.5-2070

GIS-E2-R-RCP2.6-2050

GIS-E2-R-RCP2.6-2070

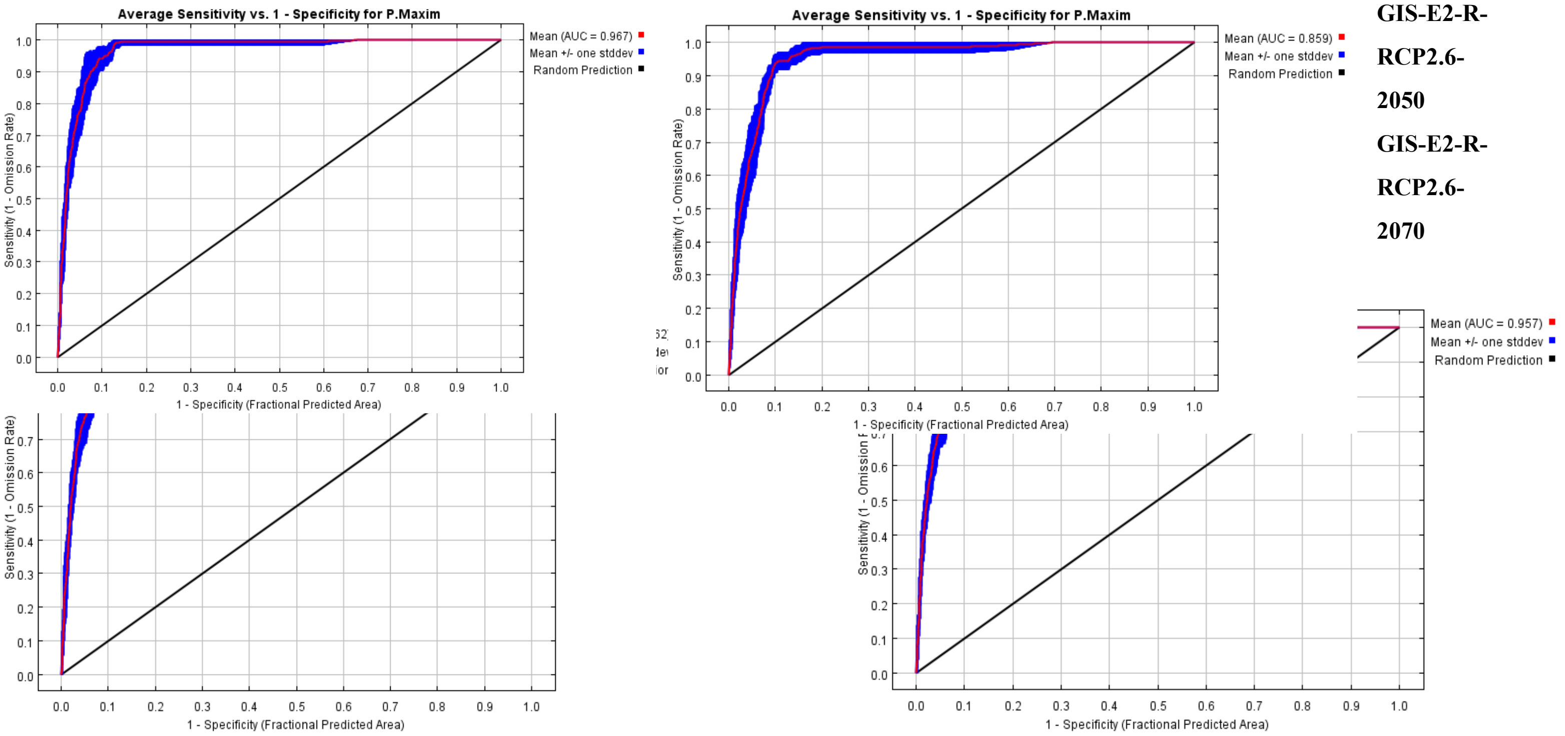

GIS-E2-R-RCP4.5-2050 **GIS-E2-R-RCP4.5-2070**

GIS-E2-R-RCP6.0-2050 **GIS-E2-R-RCP6.0-2070**

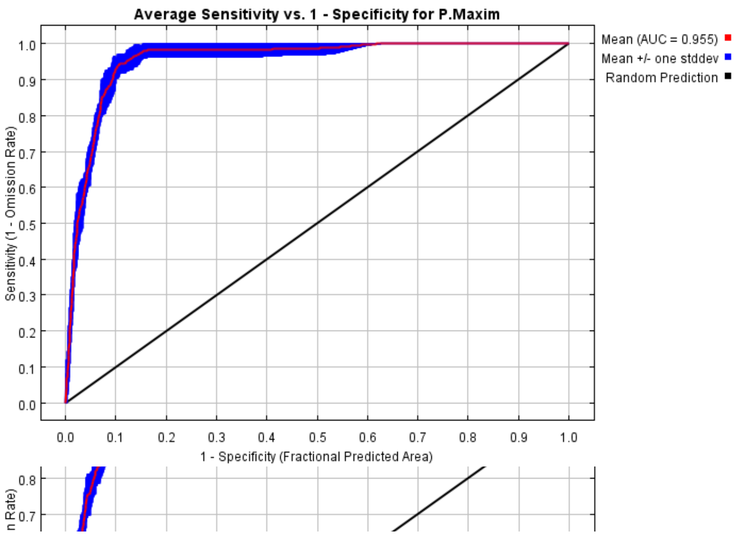

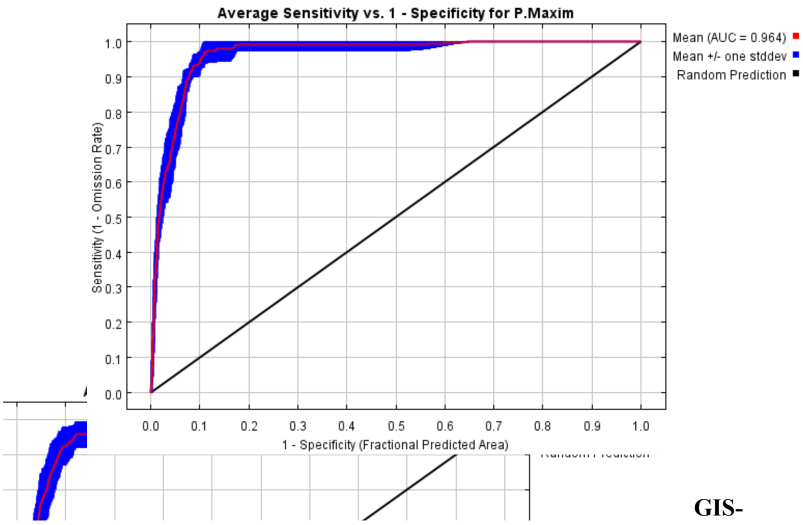

GIS-
E2-
R-

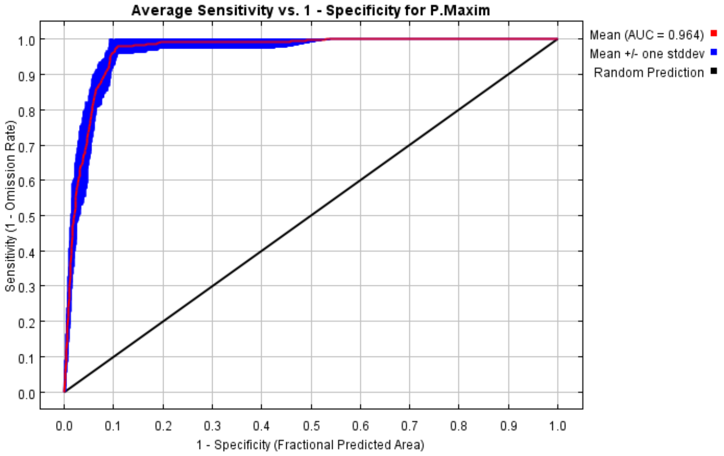

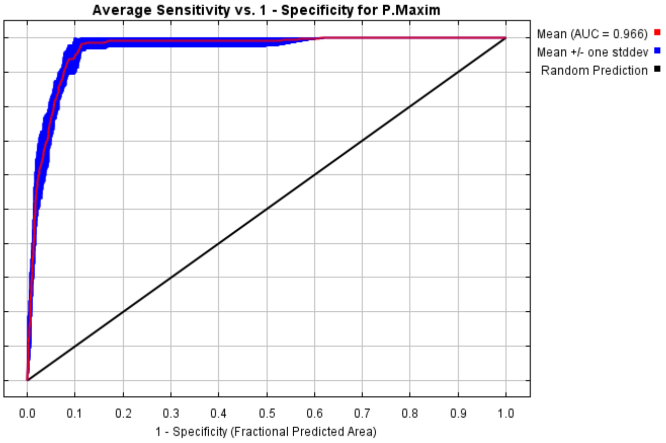

RCP8.5-2050 **GIS-E2-R-RCP8.5-2070**

HadDGEM2-ES-RCP2.6-2050 **HadDGEM2-ES-RCP2.6-2070**

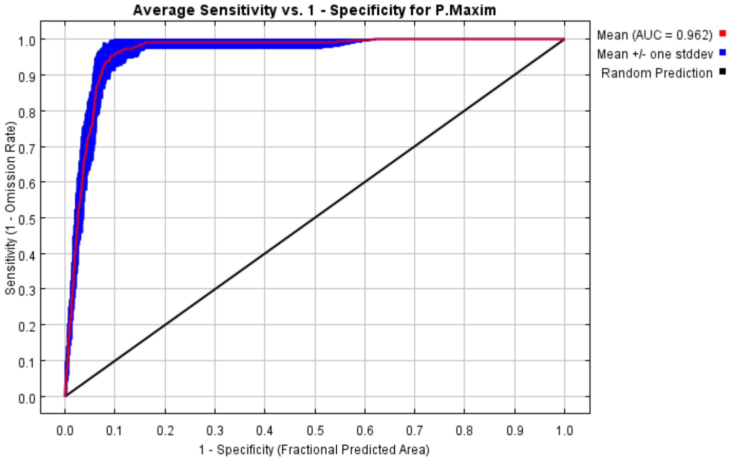

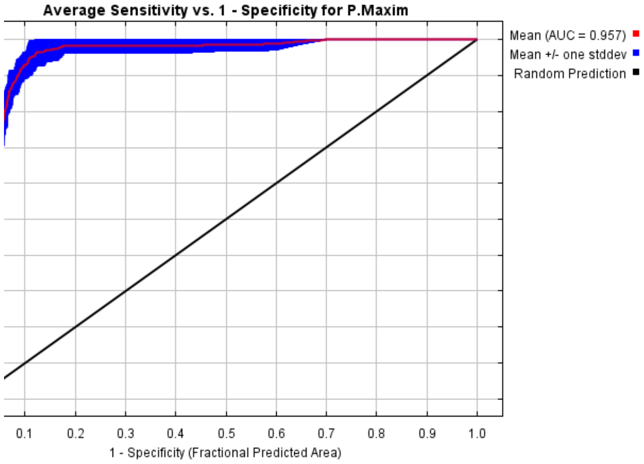

HadDGEM2-ES-RCP4.5-2050 **HadDGEM2-ES-RCP4.5-2070**

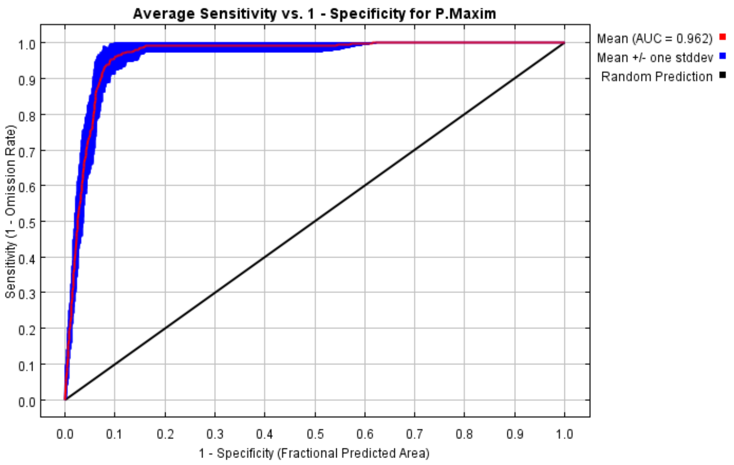

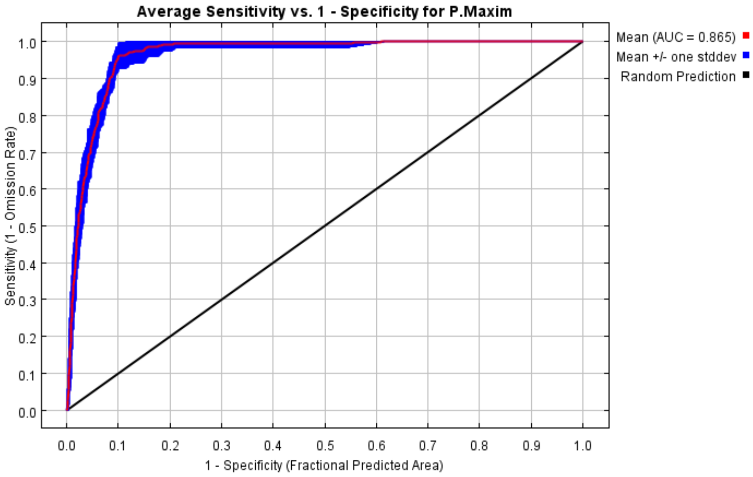

HadDGEM2-ES -RCP6.0-2050 **HadDGEM2-ES-RCP6.0-2070**

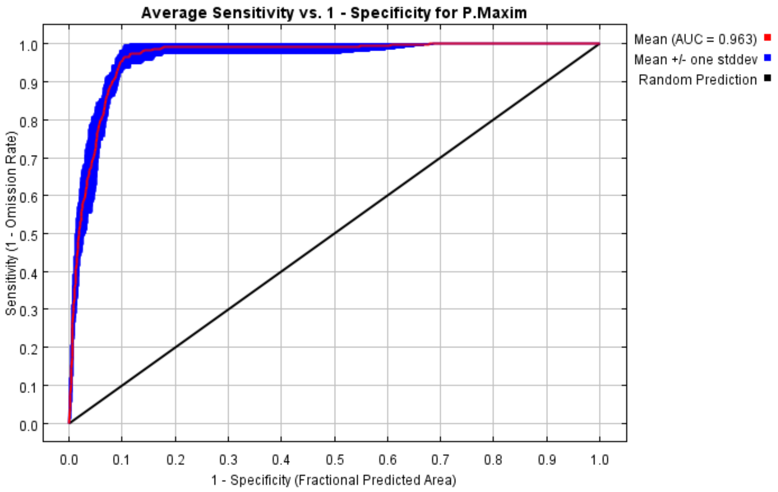

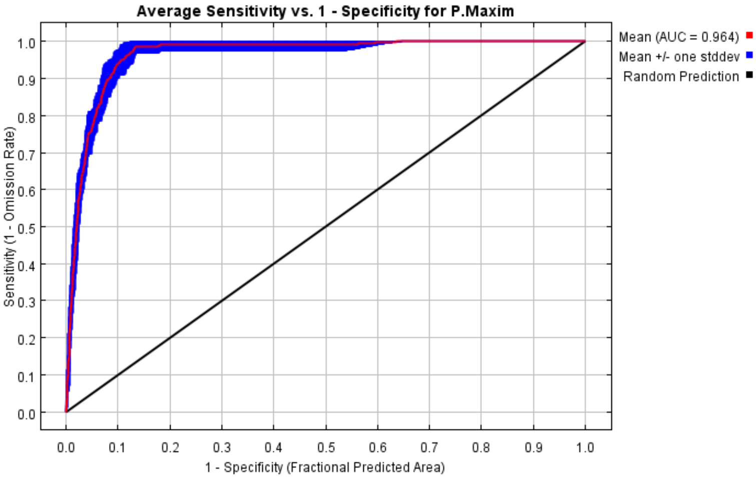

HadDGEM2-ES -RCP8.5-2050 **HadDGEM2-ES-RCP8.5-2070**

Figure S5. The results of the jackknife test of variables' contribution in modelling *Pomatosace filicula* distribution in 2050 and 2070 under four climate scenarios (RCP 2.6, RCP 4.5, RCP 6.0 and RCP 8.5) and five general circulation models(BCC-CSM1-1、CCSM4、GIS-E2-R、HadGEM2-ES and MIROC5). (The regularized training gain describes how much better the Maxent distribution fits the presence data compared to a uniform distribution. The dark blue bars indicate tHadGEM2-ESt the gain from using each variable in isolation, the light blue bars indicate the gain lost by removing the single variable from the full model, and the red bar indicates the gain using all of the variables).

MIROC5-RCP2.6-2050 **MIROC5-RCP2.6-2070**

MIROC5-RCP4.5-2050 **MIROC5-RCP4.5-2070**

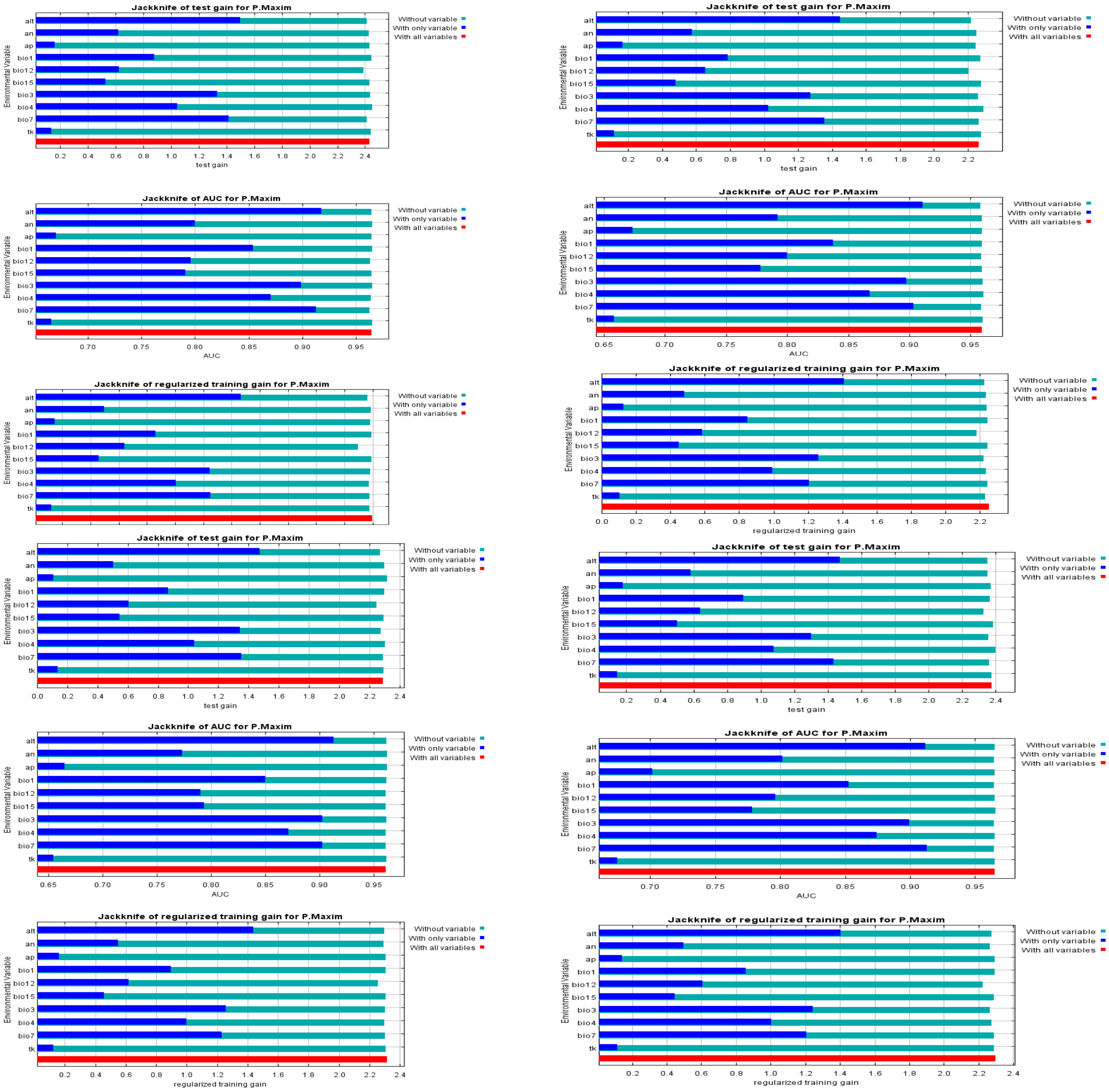

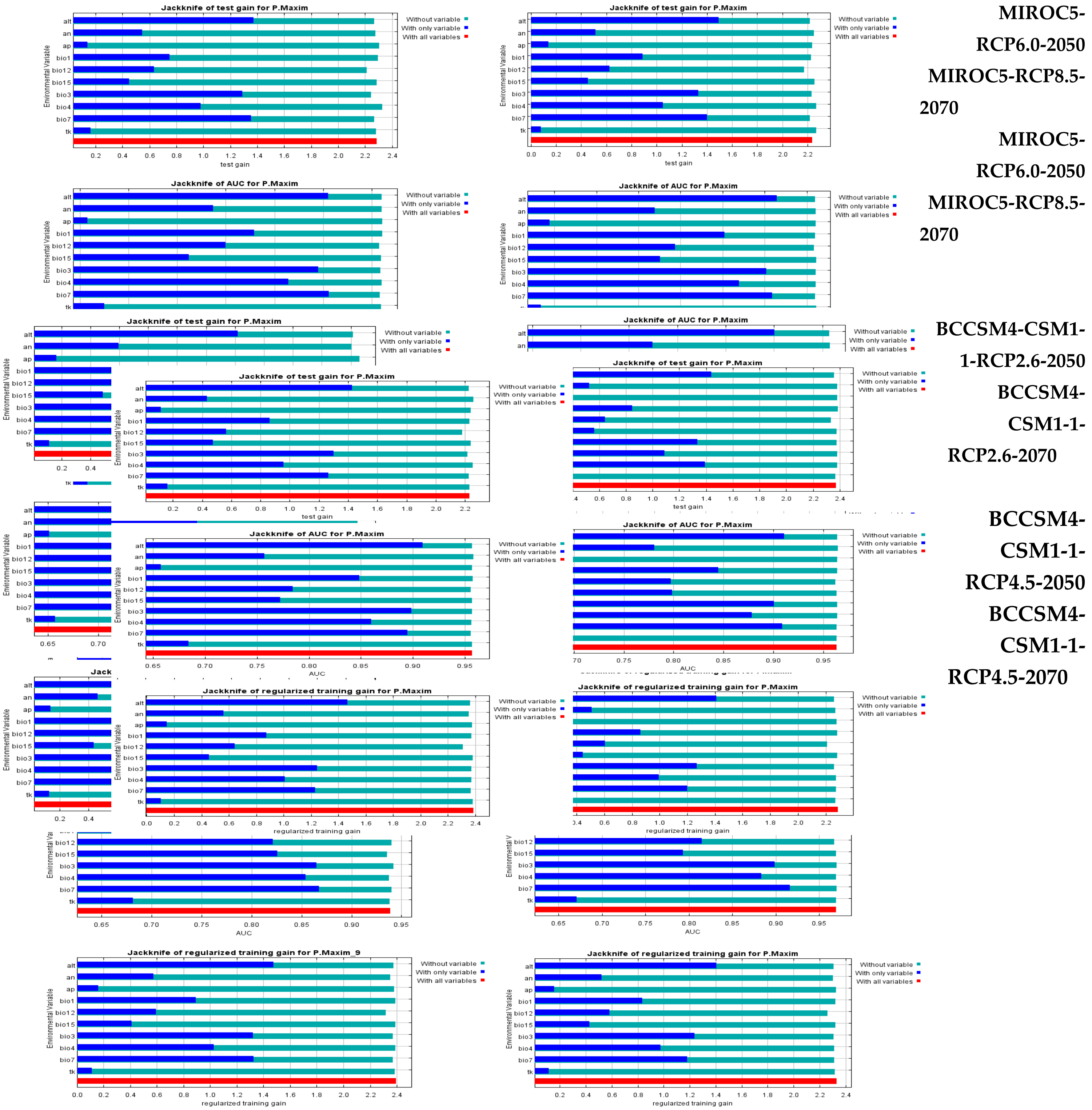
Jackknife of test gain for P.Maxim
Jackknife of AUC for P.Maxim
Jackknife of regularized training gain for P.Maxim
Jackknife of regularized training gain for P.Maxim_9
Without variable
With only variable
With all variables
Environmental Variable
test gain
AUC
regularized training gain
MIROC5-RCP6.0-2050
MIROC5-RCP8.5-2070
MIROC5-RCP6.0-2050
MIROC5-RCP8.5-2070
BCCSM4-CSM1-1-RCP2.6-2050
BCCSM4-CSM1-1-RCP2.6-2070
BCCSM4-CSM1-1-RCP4.5-2050
BCCSM4-CSM1-1-RCP4.5-2070

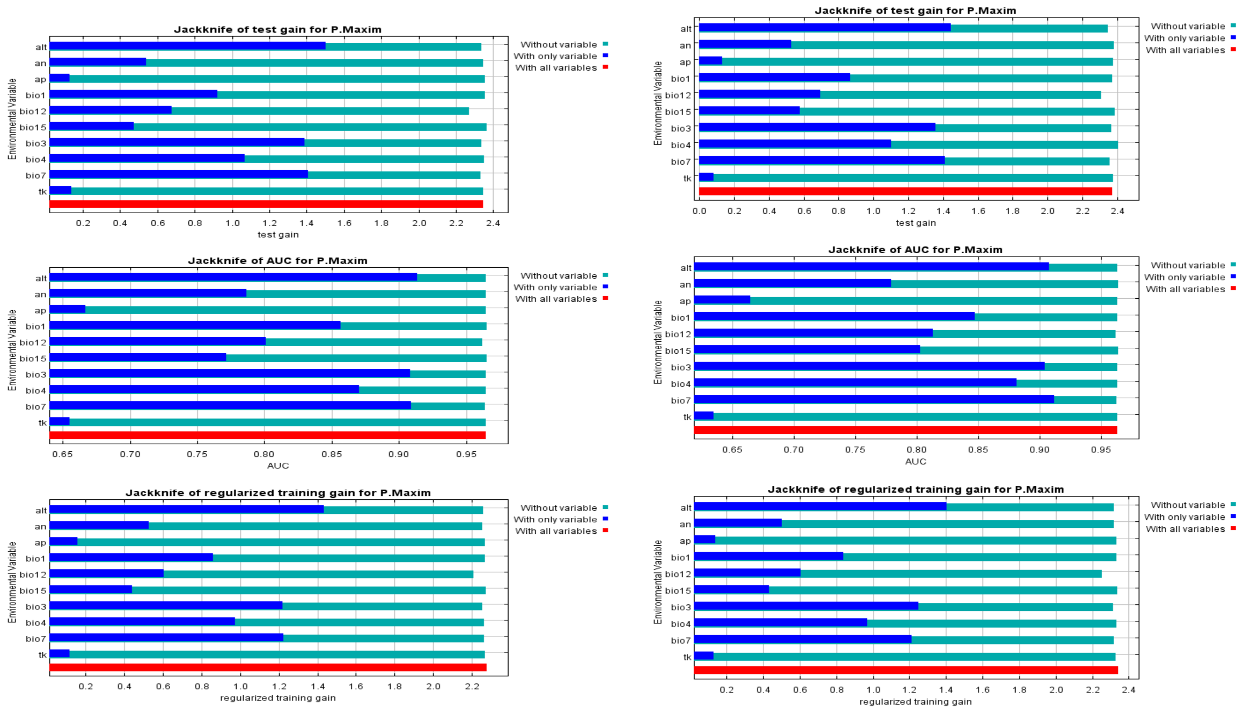

BCCSM4-CSM1-1-RCP6.0-2050

BCCSM4-CSM1-1-RCP6.0-2070

BCCSM4-CSM1-1-RCP8.5-2050

BCCSM4-CSM1-1-RCP8.5-2070

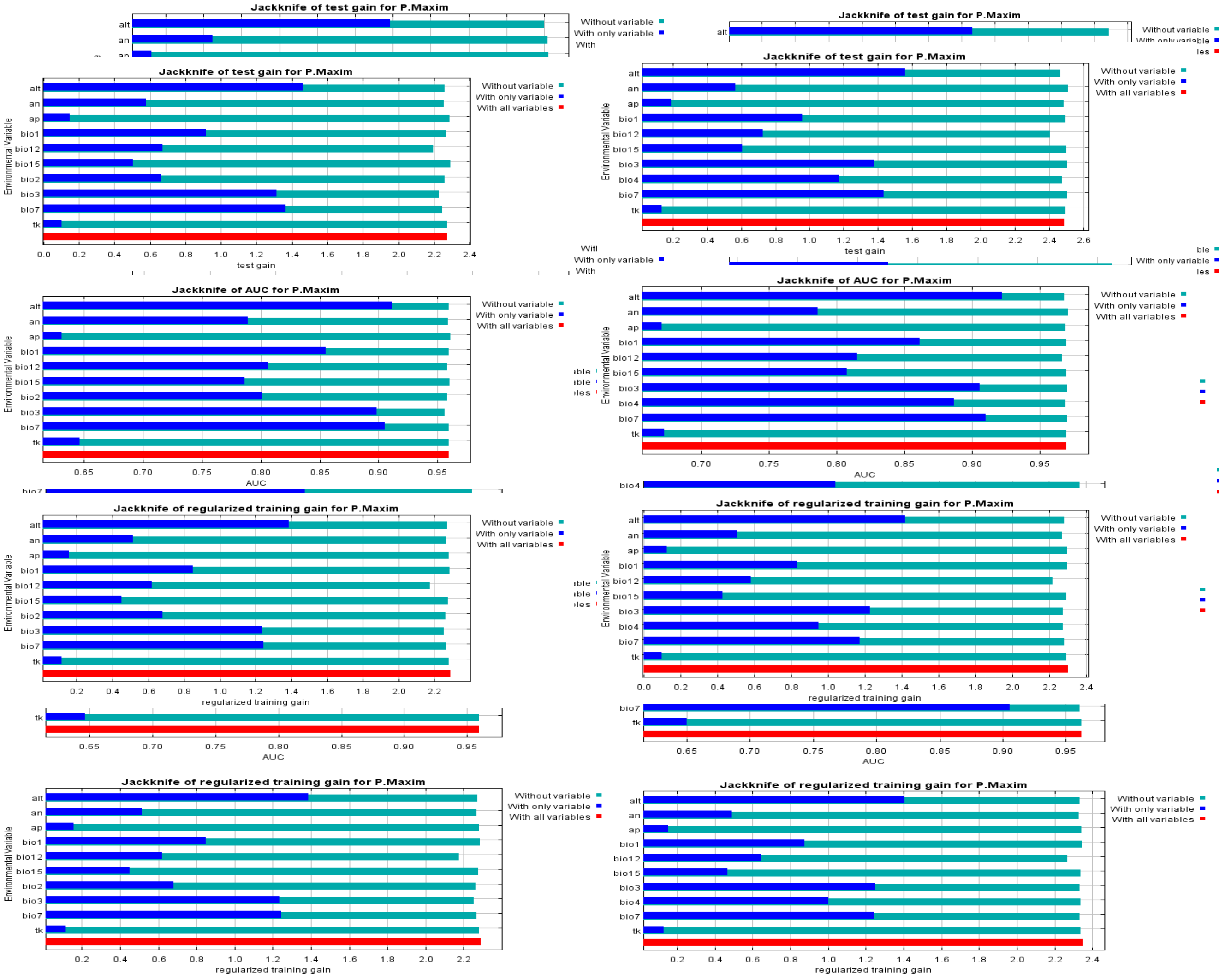

CCSM4-RCP2.6-2050

CCSM4-RCP2.6-2070

CCSM4-RCP4.5-2050

CCSM4-RCP4.5-2070

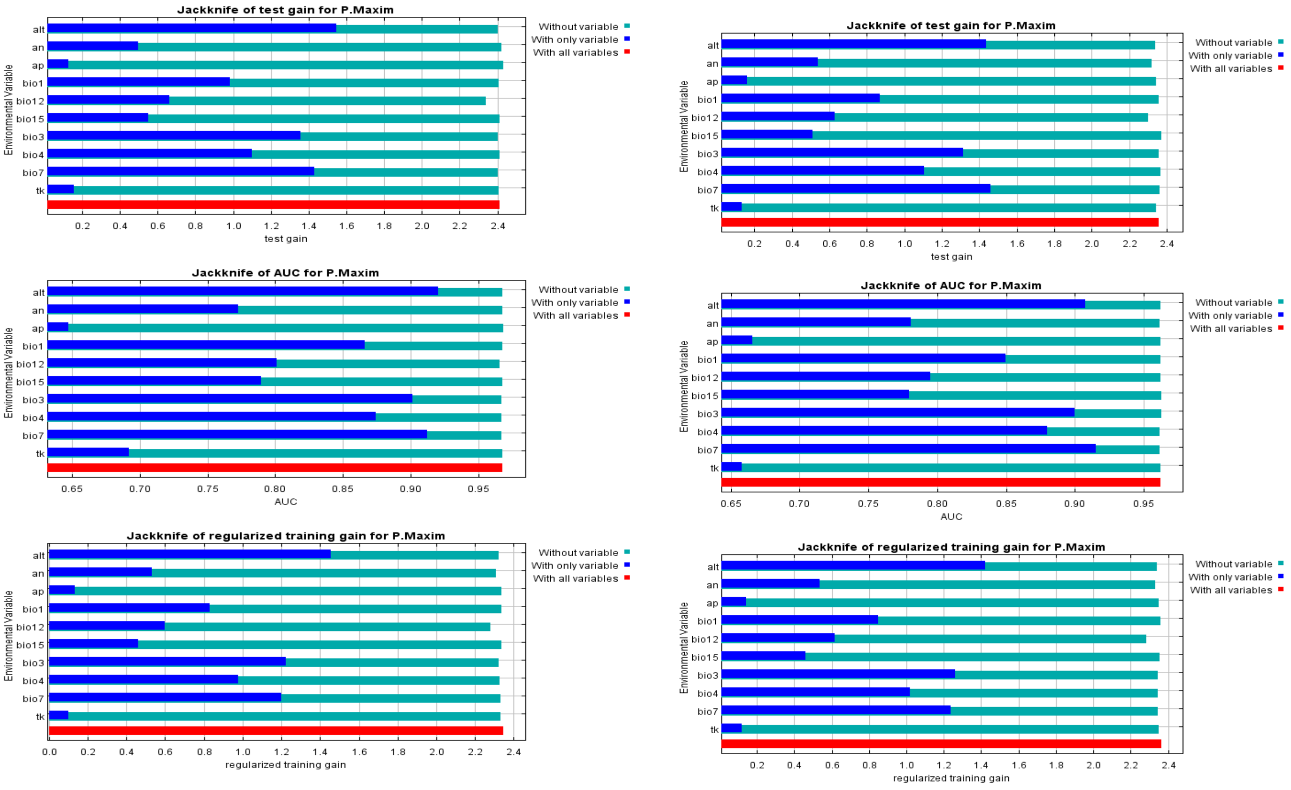

CCSM4-RCP6.0-2050 **CCSM4-RCP6.0-2070**

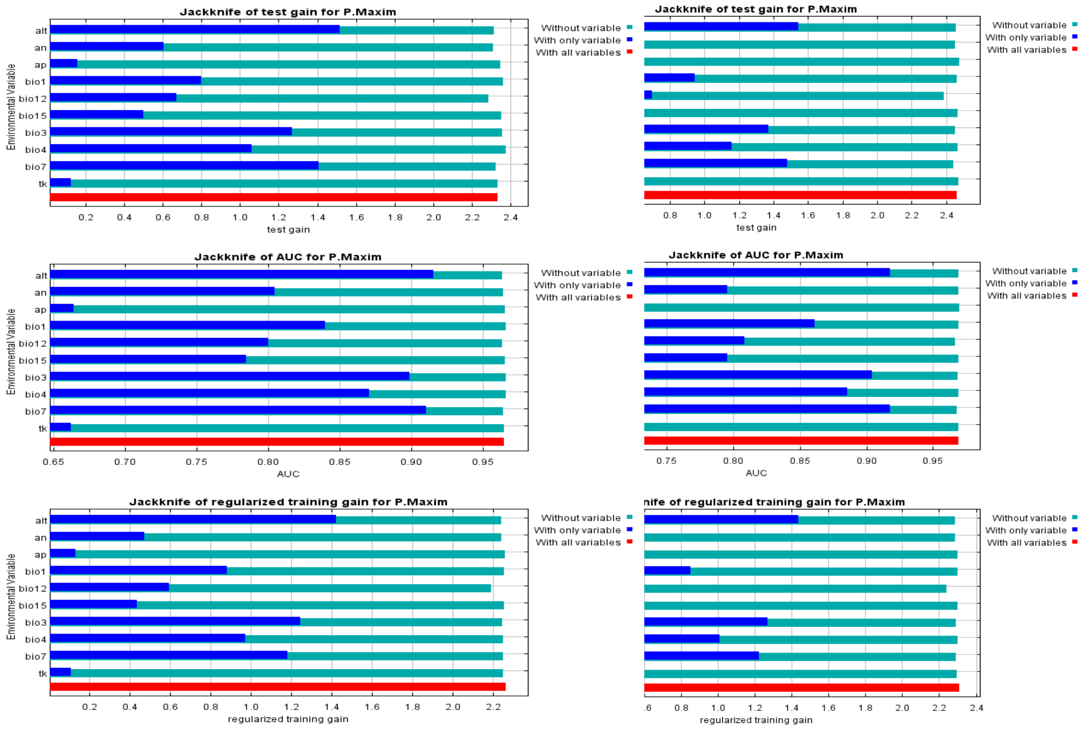

CCSM4-RCP8.5-2050
CCSM4-RCP8.5-2070

GIS-
E2-R-

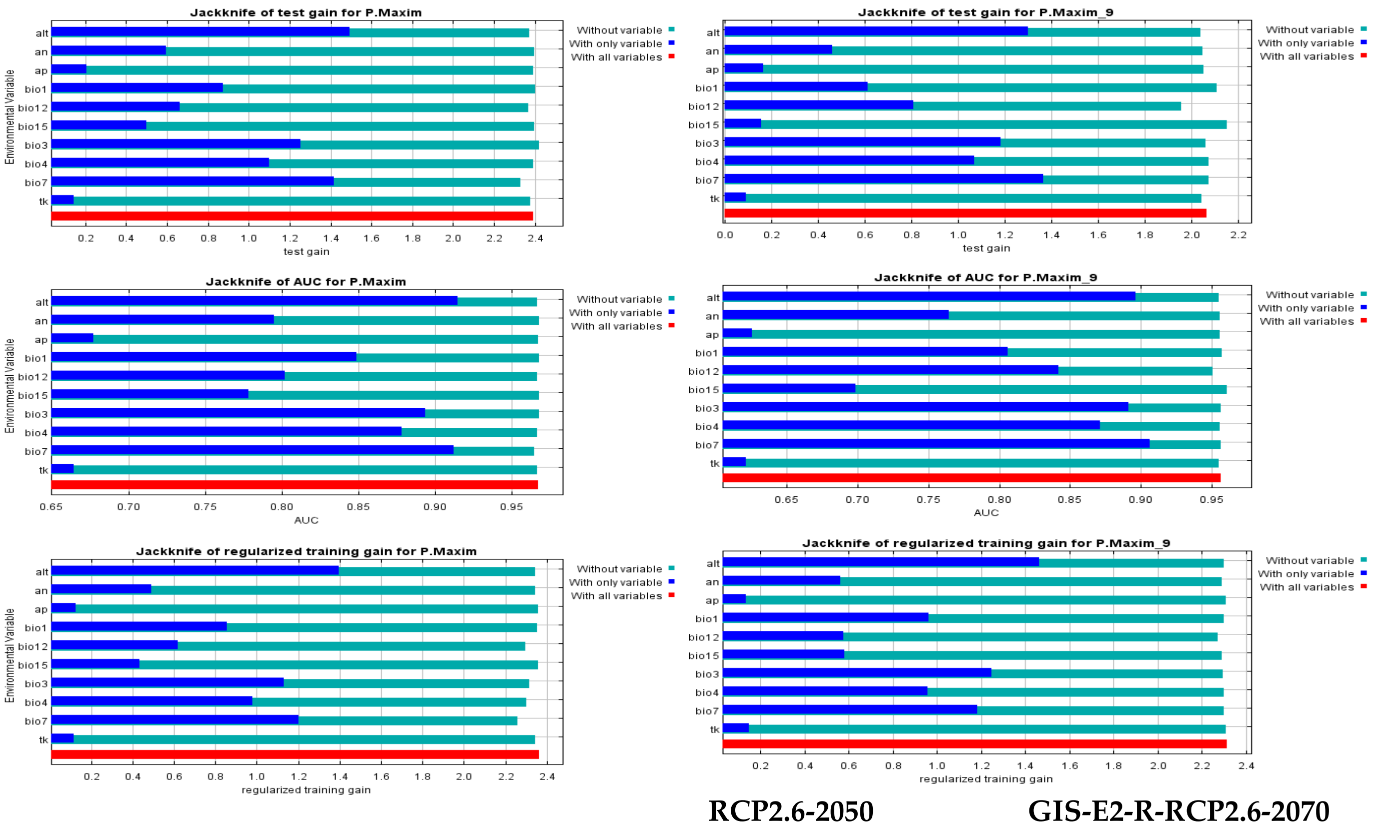

RCP2.6-2050 GIS-E2-R-RCP2.6-2070

GIS-E2-R-RCP4.5-2050 GIS-E2-R-RCP4.5-2070

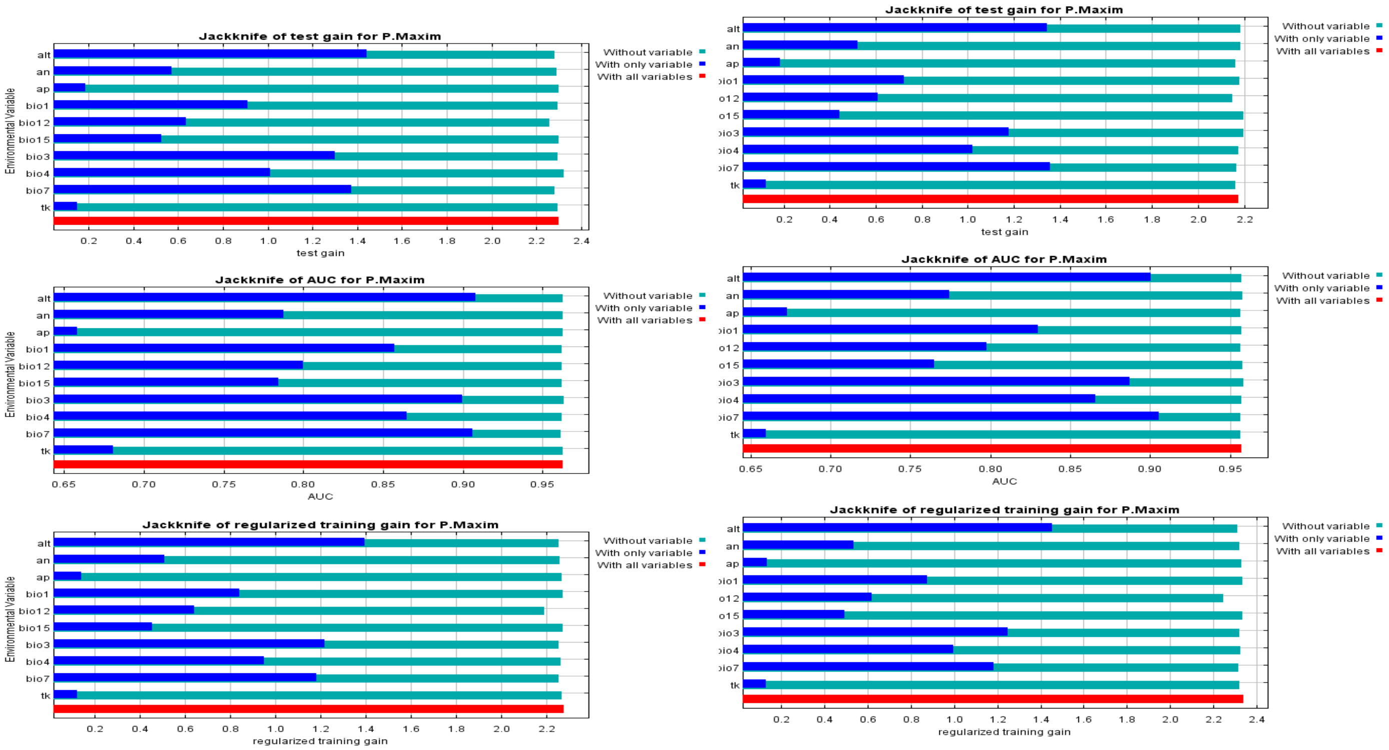

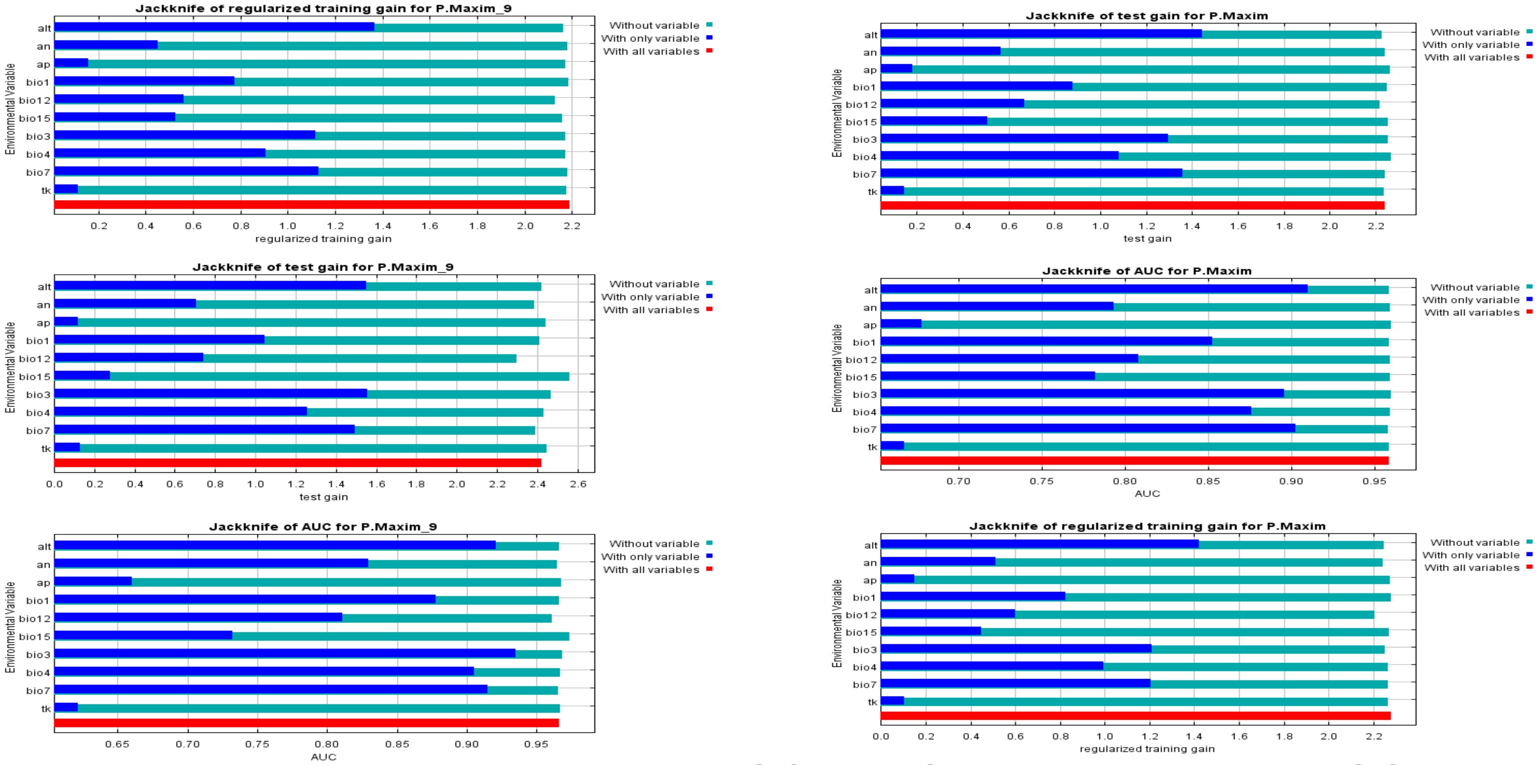

GIS-E2-R-RCP6.0-2050 **GIS-E2-R-RCP6.0-2070**

GIS-E2-R-RCP8.5-2050 **GIS-E2-R-RCP8.5-2070**

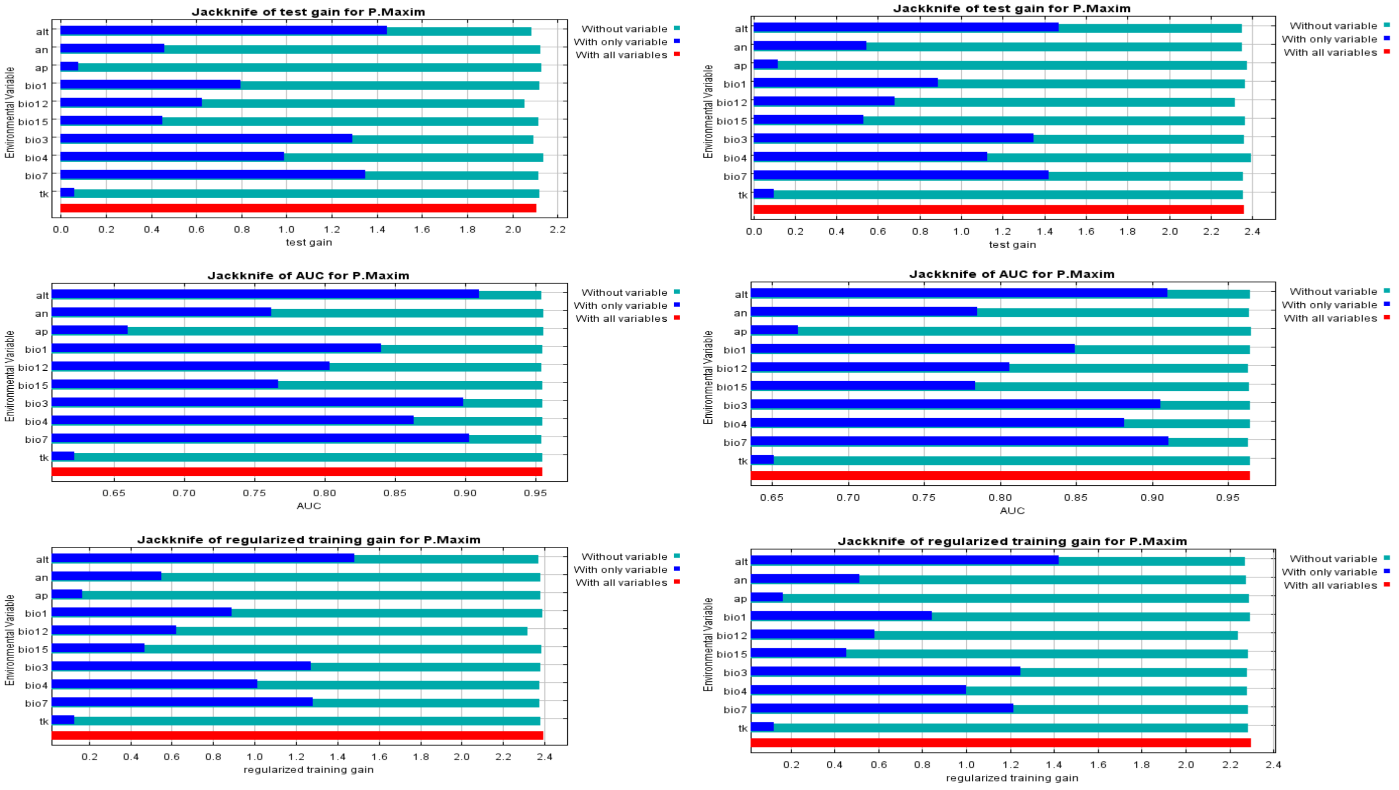

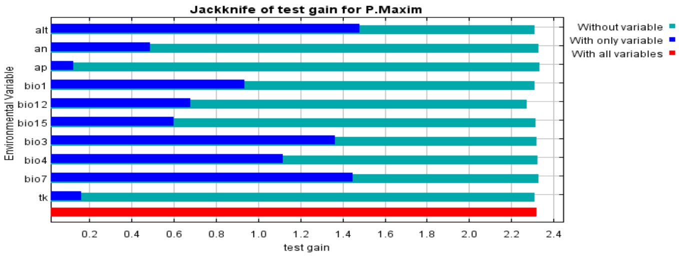

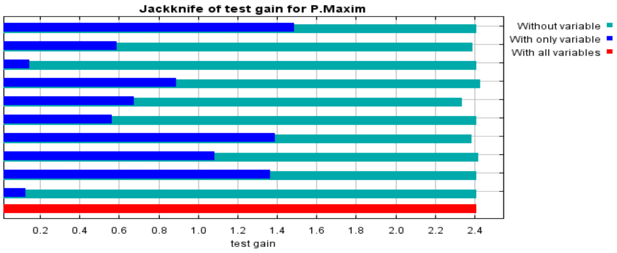

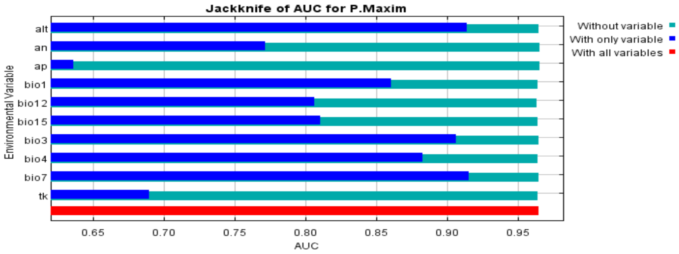

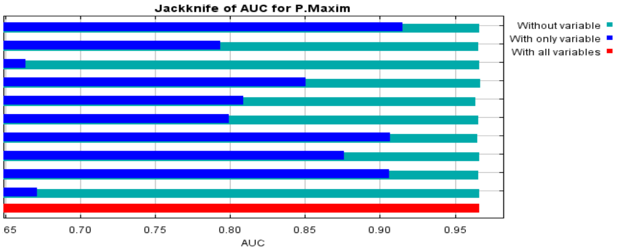

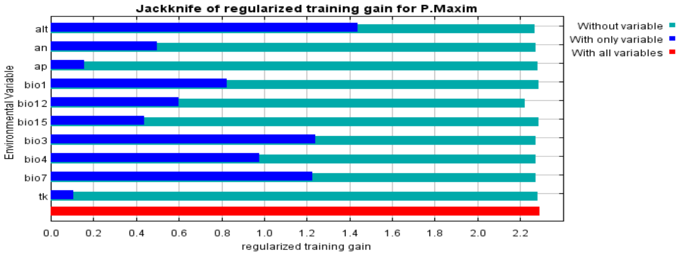

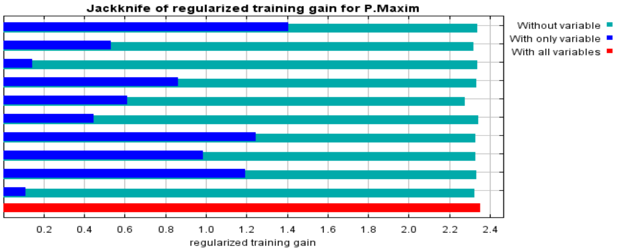

HadGEM2-ES-RCP2.6-2050 **HadGEM2-ES-RCP2.6-2070**

HadGEM2-ES-RCP4.5-2050 **HadGEM2-ES-RCP4.5-2070**

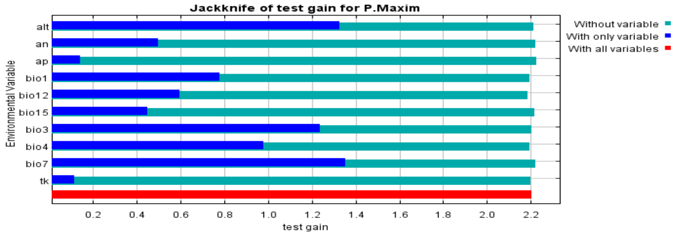

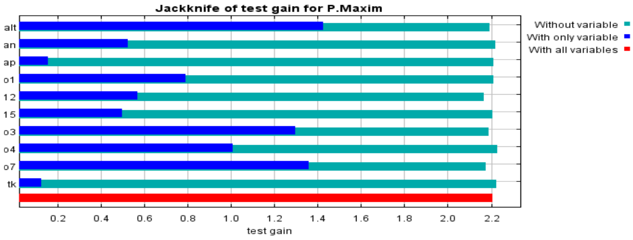

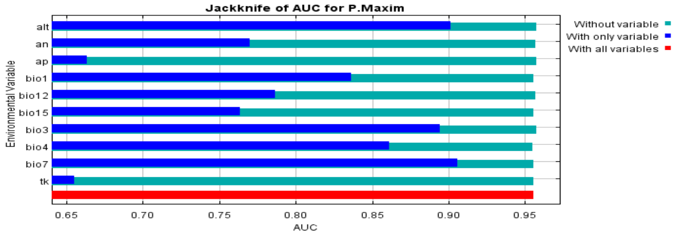

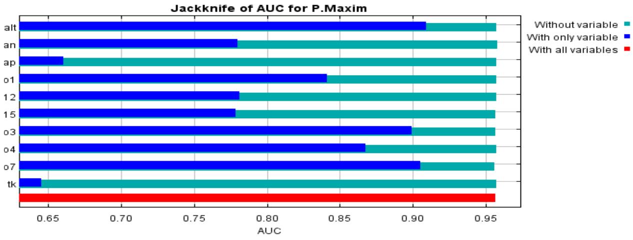

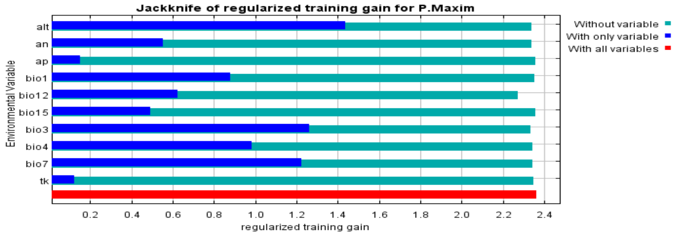

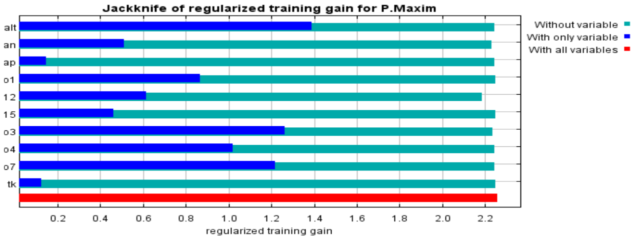

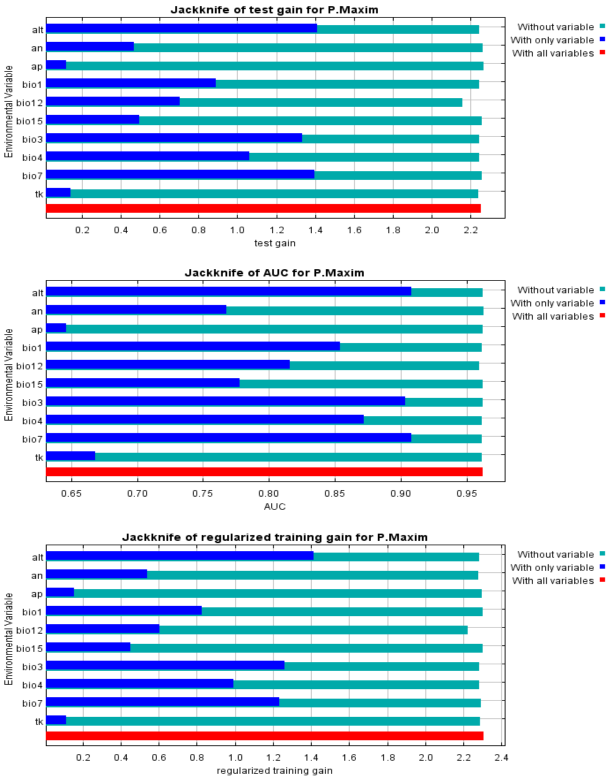

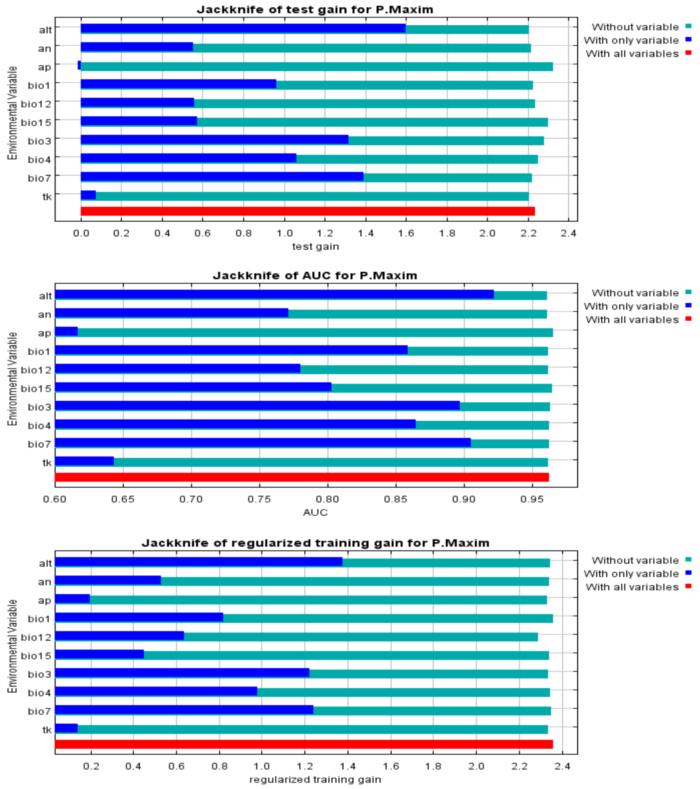

HadGEM2-ES-RCP8.5-2050 HadGEM2-ES-RCP8.5-2070 HadGEM2-ES-RCP6.0-2050
HadGEM2-ES-RCP6.0 -2050

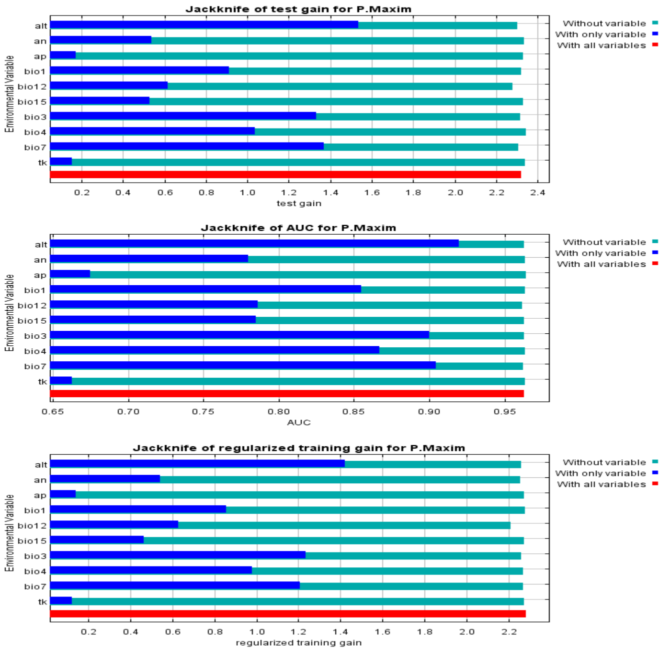

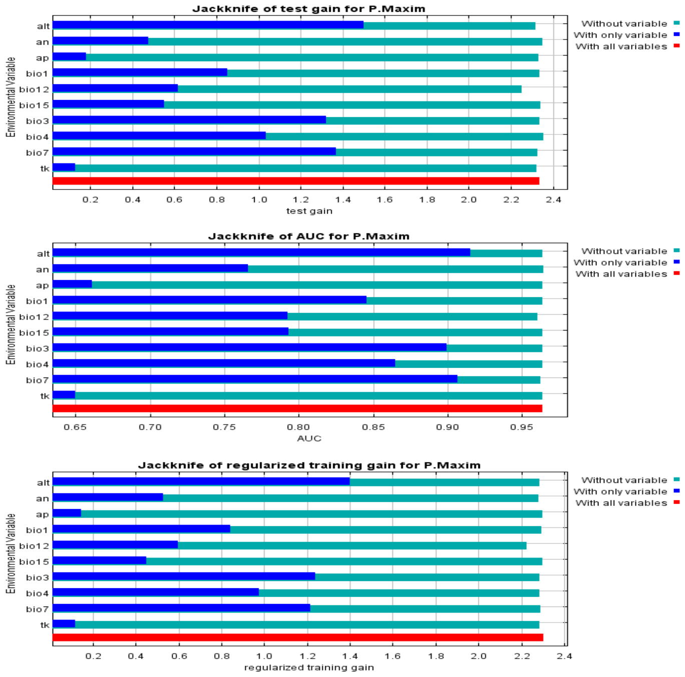

Supplement: Supplementary file 1 [file plants-11-00670-s001.zip › plants-1581232-SI.pdf]
